# Safety analysis of a live attenuated mumps vaccine in healthy adolescents in China: A phase 4, observational, open-label trial

**DOI:** 10.1371/journal.pone.0291730

**Published:** 2023-09-21

**Authors:** Weijun Hu, Ningning Jia, Weining Meng, Tiantian Zhou, Ruize Wang, Yongli Xiong, Chunfang Luan, Shaobai Zhang

**Affiliations:** 1 Immunization Programme Institute, Vaccine Clinical Evaluation Center, Shaanxi Provincial Center for Disease Control and Prevention, Xi’an, China; 2 Clinical Research and Development Center, Sinovac Biotech Co., LTD, Beijing, China; 3 Sinovac Life Sciences Co., LTD, Beijing, China; 4 Research and Development Center, Sinovac (Dalian) Vaccine Technology Co., LTD, Dalian, China; Regional Health Care and Social Agency of Lodi, ITALY

## Abstract

Mumps is an acute infectious disease, which was well controlled in the past, but recently it has resurged in some areas. This study aimed to evaluate the safety profile of the live attenuated mumps vaccine after large-scale vaccination. We conducted an observational, open-label phase 4 trial in Shaanxi, China from October 2020 to March 2021. Eligible participants were freshmen of junior high school who were not above 14 years old. Adverse events following immunization (AEFI) monitoring was carried out by active and passive surveillance. Safety follow-ups were conducted during the study participation. Overall, 10057 subjects were enrolled in the active surveillance analysis. A total of 214 subjects reported adverse reactions with an incidence of 2.13% (214/10057). Most adverse reactions were grade 1, and the incidence of grade 1 adverse reactions was 1.44% (145/10057); 0.60% for grade 2 (60/10057); and 0.09% for grade 3 (9/10057). The majority of adverse reactions were solicited (1.73%, 174/10057). Injection-site pain was the most frequently reported local adverse reaction (0.71%, 71/10057), followed by redness (0.29%, 29/10057). The most common systemic adverse reactions were nausea (0.19%, 19/10057) and fever (0.16%, 16/10057). For passive AEFI surveillance, 57 AEFI cases were reported, with an incidence of 19.28/100000 (57/287608). And most AEFI cases were common adverse reactions (66.67%, 38/57). In total, the live attenuated mumps vaccine evaluated in this trial has a favorable safety profile and can be used for large-scale inoculation.

## Introduction

Mumps is an acute respiratory infectious disease caused by the mumps virus, which is characterized by inflammation and swelling of unilateral or bilateral parotid glands and can subsequently lead to encephalitis, orchitis, or meningitis [1–3]. Mumps spreads rapidly in dense susceptible populations and is prone to outbreaks in places where children are concentrated, such as schools and kindergartens [4]. As the only natural host of mumps virus, before widespread use of mumps vaccine, almost everyone had serological evidence of mumps infection before the age of 15 [5]. Therefore, most countries regard mumps as one of the major communicable diseases in their childhood immunization programs [6]. Vaccination with a live attenuated mumps vaccine is the most effective way to prevent mumps, and the incidence of mumps has decreased substantially in areas where large-scale vaccination has been carried out. For example, on mainland China, the incidence of mumps has declined from 100–1000/100000 in the 1970s to 21.48/100000 in 2019 [7,8].

In recent years, however, this well-controlled disease has been reported to occasionally re-emerge in some countries such as the United States, Canada, and France, which have well-established vaccination programs [9–11]. The underlying reasons are complex, but a growing body of research suggests that mumps vaccine-induced immunity wanes over time [12–14]. Hence, it is necessary to take some innovative measures, such as developing a new effective mumps vaccine or changing the current mumps vaccine immunization strategy, so as to achieve complete control of the mumps epidemic. Notably, the World Health Organization (WHO) has recommended the introduction of a second dose of measles-mumps-rubella (MMR) vaccine into the vaccination program to ensure lasting immunity against mumps.

The live attenuated mumps vaccine is made from the S79 strain, which included two subtypes, S79 major and S79 minor. The full genome sequences of the two subtypes and their corresponding Jeryl-Lynn strain are highly conserved, with homologies of 99.7% and 100%, respectively. The F protein and HN protein, which induce protective antibodies, showed no genetic differences [15]. In China, mumps-containing vaccines were included in the country’s Expanded Program on Immunization (EPI) in 2008 [16,17]. Although the incidence of mumps was greatly reduced after the introduction of vaccination against mumps, it has remained at a level around 20/100000 since 2004 [18]. In recent years, mumps ranks among the top five notifiable infectious diseases (out of a total of 12) in Class C reporting. In 2019, approximately 300,000 mumps cases were reported, children and adolescents under the age of 15 accounting for over 80%. There were two peak incidences: one was at the age of 5–9 and the other was at the age of 10–15 [19,20]. The study aimed to evaluate the safety of the live attenuated mumps vaccine produced by Sinovac (Dalian) after large-scale applications, and accumulate safety data for the application of mumps vaccine.

## Materials and methods

### Study design and participants

This phase 4, open-label, observational study was conducted in Shaanxi Province, China, from October 2020 to March 2021. Safety observations after vaccination were carried out by a combination of active surveillance and passive surveillance. Active surveillance collected safety data by selecting some vaccination units in four cities of Shaanxi Province (Baoji City, Xianyang City, Yan’an City, and Hanzhong City) as the research sites, while passive surveillance collected Adverse Events Following Immunization (AEFI) data after vaccination from the "Chinese National AEFI Information System (CNAEFIS)".

Eligible participants were freshmen of junior high school who were not above 14 years old and were able to complete safety follow-ups and to adhere to research procedures. Written informed consent was obtained from the subject’s parents or guardians before enrollment. This study was approved by the ethical review committee of the Shaanxi Provincial Center for Disease Control and Prevention (2020-001-08). The trial was registered with ClinicalTrials.gov (NCT05145166).

### Study vaccine

The live attenuated mumps vaccine developed by Sinovac (Dalian) Vaccine Technology Co., Ltd, was manufactured by inoculating primary chicken embryo cells with the mumps virus S79 strain, culturing and harvesting the virus solution, and freeze-dried with suitable stabilizer. Each dose contains not less than 10^3.7^ CCID_50_ of live mumps virus in a total volume of 0.5 mL/vial. The investigational mumps vaccine (lot 202001001, expiry 4 July 2021; lot 202001002, expiry 5 July 2021; lot 202002014, expiry 16 August 2021; and lot 202002016, expiry 20 August 2021) was tested by the National Institutes for Food and Drug Control (NIFDC) and confirmed to adhere to the necessary specifications.

### Safety assessment

For active surveillance, immediate adverse events were observed on the study site during 30 minutes following vaccination. Face-to-face or telephone visits were assigned on the 14th day and 30th day to collect solicited and unsolicited adverse events after vaccination. Solicited adverse events are defined as symptoms specified in the protocol occurring within 14 days after vaccination. Unsolicited adverse events refer to symptoms specified in the protocol occurring from Day 15 to Day 30, or other symptoms which were not specified in the protocol occurring within 30 days after vaccination. Solicited systemic adverse events included nausea, vomiting, diarrhea, fever, allergic reaction, fatigue, skin and mucosa abnormality, cough, headache, decreased appetite, and muscle pain; solicited local adverse events included rash, pain, swelling, pruritus, redness, and induration. Any adverse events were graded according to the guidelines issued by the National Medical Products Administration (NMPA) [21]. ‘Adverse events’ refer to any medical adverse events that occurred after clinical trial subjects received the investigational vaccine, which were not necessarily causally related to the investigational vaccine, while ‘adverse reactions’ were usually related to the vaccines or vaccination. The causal relationship between adverse events and vaccination was judged by the study staff. Moreover, if a participant reported two or more adverse events/ adverse reactions, the number of the participants with adverse events/ adverse reactions will be counted as one. Safety assessment was conducted based on the safety set.

For passive surveillance, AEFI data were extracted from the CNAEFIS within 3 months after vaccination. AEFI refers to a reaction or event that occurs after vaccination and is suspected to be related to vaccination [22]. It can be classified into five types: adverse reaction (common adverse reaction, rare adverse reaction), vaccine quality event, program error, coincidental event, and psychogenic reaction. Each reported AEFI record should be investigated by the researcher according to China’s national AEFI guidelines [23].

### Statistical analysis

Statistical analysis was performed using SAS version 9.4 (SAS Institute Inc, Cary, North Carolina, USA). Categorical variables were reported as frequency and percentage, continuous variables were reported as means and standard deviations (SD). The One-Way ANOVA or Kruskal-Wallis test was used for comparison of continuous data, and χ2 test /Fisher’s exact test was used for comparison of categorical data. A 2-side p-value of less than 0.05 was considered as statistically significant.

## Results

### Study subjects

Overall, 10057 subjects were recruited and included in the active surveillance analysis, including 2557 in Baoji City, 2500 in Hanzhong City, 2500 in Xianyang City, and 2500 in Yan’an City. The age and sex characteristics of the subjects were presented in Table 1, showing a median age of 12.12 years (SD 0.74) and a sex ratio of 1.16:1. As for passive AEFI surveillance, a total of 287608 subjects were included in the safety analysis.

**Table 1:** Baseline characteristics of the study participants.

	Baoji City	Hanzhong City	Xianyang City	Yan’an City	Total
No. of participants	2557	2500	2500	2500	10057
Age, years	11.90 ± 0.72	12.13 ± 0.71	12.23 ± 0.71	12.22 ± 0.78	12.12 ± 0.74
Sex, n (%)					
Male	1378 (53.89)	1299 (51.96)	1355 (54.20)	1363 (54.52)	5395 (53.64)
Female	1179 (46.11)	1201 (48.04)	1145 (45.80)	1137 (45.48)	4662 (46.36)

Data are no. (%) of participants or mean ± SD.

### Safety

The overall profiles of adverse events after vaccination were summarized in Table 2. In total, 852 subjects reported adverse events, with an incidence of 8.47% (852/10057). The overall adverse reactions rate was 2.13% (214/10057), of which the adverse reaction rates in Baoji City, Hanzhong City, Xianyang City, and Yan’an City were 3.68% (94/2557), 0.84% (21/2500), 1.52% (38/2500) and 2.44% (61/2500), respectively. There was a statistically significant difference in the adverse reaction rates between these four cities (*p* < 0.001).

**Table 2:** Overall profiles of adverse events after vaccination.

	Baoji City (n = 2557)	Hanzhong City (n = 2500)	Xianyang City (n = 2500)	Yan’an City (n = 2500)	Total (N = 10057)	*P* value
**Overall adverse events**	308 (12.05)	195 (7.80)	57 (2.28)	292 (11.68)	852 (8.47)	<0.001
**Vaccine-related adverse events (adverse reactions)**	94 (3.68)	21 (0.84)	38 (1.52)	61 (2.44)	214 (2.13)	<0.001
**Vaccine-unrelated adverse events**	219 (8.56)	176 (7.04)	19 (0.76)	249 (9.96)	663 (6.59)	<0.001

Data are no. (%) of participants. Adverse reactions were considered as vaccine-related adverse events. Participants could have more than one adverse event /reaction.

**Fig 1:**
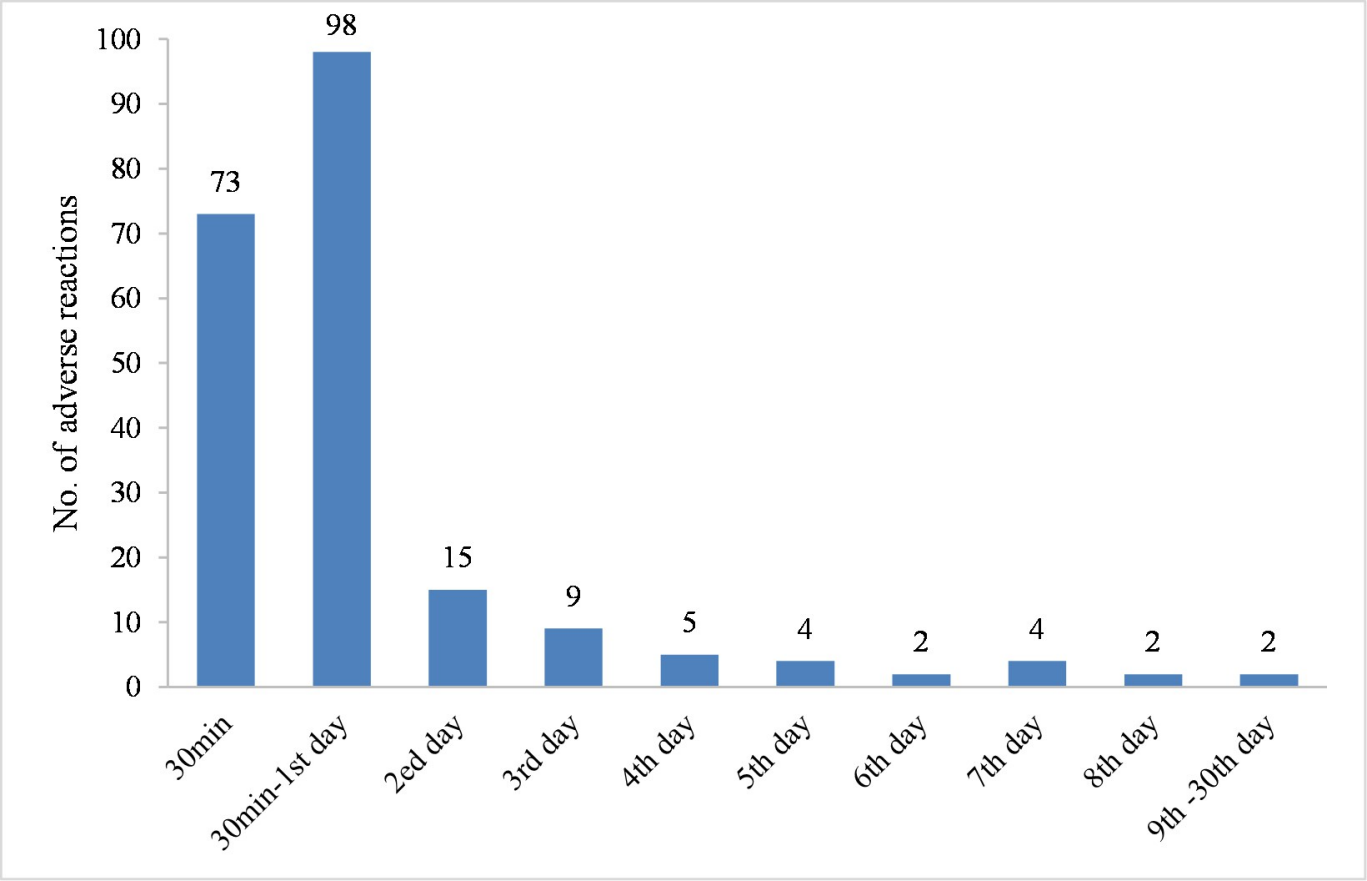
Number of adverse reactions at different time points after vaccination.

In terms of adverse reaction onset time, 74 (0.74%) of 10057 participants reported immediate adverse reactions based on clinical observation for 30 minutes after inoculation. All immediate adverse reactions were non-serious and participants recovered without any complications. In addition, almost 80% (172/214) of adverse reactions occurred within 1 day after vaccination. The distribution of adverse reactions onset time was shown in Fig 1.

As shown in Table 3, the majority of adverse reactions were solicited (1.73%, 174/10057), including 137 (1.36%) with local reactions and 51 (0.51%) with systemic reactions. In addition, most adverse reactions were grade 1 in intensity, and the incidence of grade 1 adverse reactions was 1.44% (145/10057); 0.60% for grade 2 (60/10057); and 0.09% for grade 3 (9/10057). There were no grade 4 and above reported.

**Table 3:** Summary of adverse reactions by severity.

Adverse reactions	Baoji City (n = 2557)	Hanzhong City (n = 2500)	Xianyang City (n = 2500)	Yan’an City (n = 2500)	Total (N = 10057)
**Overall**	94 (3.68)	21 (0.84)	38 (1.52)	61 (2.44)	214 (2.13)
Grade 1	70 (2.74)	19 (0.76)	27 (1.08)	29 (1.16)	145 (1.44)
Grade 2	24 (0.94)	1 (0.04)	11 (0.44)	24 (0.96)	60 (0.60)
Grade 3	0 (0.00)	1 (0.04)	0 (0.00)	8 (0.32)	9 (0.09)
**Solicited reactions**	67 (2.62)	19 (0.76)	37 (1.48)	51 (2.04)	174 (1.73)
Grade 1	57 (2.23)	18 (0.72)	27 (1.08)	29 (1.16)	131 (1.30)
Grade 2	10 (0.39)	1 (0.04)	10 (0.40)	16 (0.64)	37 (0.37)
Grade 3	0 (0.00)	0 (0.00)	0 (0.00)	6 (0.24)	6 (0.06)
**Local reactions**	57 (2.23)	8 (0.32)	35 (1.40)	37 (1.48)	137 (1.36)
Grade 1	50 (1.96)	8 (0.32)	27 (1.08)	27 (1.08)	112 (1.11)
Grade 2	7 (0.27)	0 (0.00)	8 (0.32)	6 (0.24)	21 (0.21)
Grade 3	0 (0.00)	0 (0.00)	0 (0.00)	4 (0.16)	4 (0.04)
**Systemic reactions**	11 (0.43)	11 (0.44)	4 (0.16)	25 (1.00)	51 (0.51)
Grade 1	7 (0.27)	10 (0.40)	2 (0.08)	11 (0.44)	30 (0.30)
Grade 2	4 (0.16)	1 (0.04)	2 (0.08)	12 (0.48)	19 (0.19)
Grade 3	0 (0.00)	0 (0.00)	0 (0.00)	2 (0.08)	2 (0.02)
**Unsolicited reactions**	28 (1.10)	4 (0.16)	2 (0.08)	15 (0.60)	49 (0.49)
Grade 1	13 (0.51)	3 (0.12)	1 (0.04)	0 (0.00)	17 (0.17)
Grade 2	15 (0.59)	0 (0.00)	1 (0.04)	13 (0.52)	29 (0.29)
Grade 3	0 (0.00)	1 (0.04)	0 (0.00)	2 (0.08)	3 (0.03)

Data are no. (%) of participants.

The incidences of local and systemic adverse reactions are presented in Table 4. The most frequently reported solicited local adverse reactions were pain at injection site (0.71%, 71/10057), redness (0.29%, 29/10057), swelling (0.17%, 17/10057), and pruritus (0.17%, 17/10057). The most commonly reported solicited systemic adverse reactions were nausea (0.19%, 19/10057), fever (0.16%, 16/10057), and headache (0.08%, 8/10057). Some of the commonly reported unsolicited adverse reactions were upper respiratory infection, dizziness, abdominal pain, joint pain, and lymphadenitis (S1 Table).

**Table 4:** Local and systemic adverse reaction symptoms after vaccination.

Adverse reactions	Baoji City (n = 2557)	Hanzhong City (n = 2500)	Xianyang City (n = 2500)	Yan’an City (n = 2500)	Total (N = 10057)
**Solicited**	67 (2.62)	19 (0.76)	37 (1.48)	51 (2.04)	174 (1.73)
** Local**	57 (2.23)	8 (0.32)	35 (1.40)	37 (1.48)	137 (1.36)
Rash	3 (0.12)	0 (0.00)	0 (0.00)	7 (0.28)	10 (0.10)
Pain	30 (1.17)	5 (0.20)	19 (0.76)	17 (0.68)	71 (0.71)
Swelling	3 (0.12)	0 (0.00)	4 (0.16)	10 (0.40)	17 (0.17)
Pruritus	7 (0.27)	0 (0.00)	6 (0.24)	4 (0.16)	17 (0.17)
Redness	14 (0.55)	2 (0.08)	10 (0.40)	3 (0.12)	29 (0.29)
Induration	0 (0.00)	1 (0.04)	3 (0.12)	6 (0.24)	10 (0.10)
** Systemic**	11 (0.43)	11 (0.44)	4 (0.16)	25 (1.00)	51 (0.51)
Nausea	4 (0.16)	2 (0.08)	0 (0.00)	13 (0.52)	19 (0.19)
Vomiting	2 (0.08)	1 (0.04)	0 (0.00)	3 (0.12)	6 (0.06)
Diarrhea	0 (0.00)	1 (0.04)	0 (0.00)	1 (0.04)	2 (0.02)
Fever	4 (0.16)	6 (0.24)	2 (0.08)	4 (0.16)	16 (0.16)
Fatigue	0 (0.00)	0 (0.00)	0 (0.00)	2 (0.08)	2 (0.02)
Skin and mucosa abnormality	1 (0.04)	0 (0.00)	0 (0.00)	1 (0.04)	2 (0.02)
Cough	3 (0.12)	0 (0.00)	0 (0.00)	2 (0.08)	5 (0.05)
Headache	2 (0.08)	2 (0.08)	2 (0.08)	2 (0.08)	8 (0.08)
Decreased appetite	0 (0.00)	1 (0.04)	0 (0.00)	0 (0.00)	1 (0.01)
Muscle pain	0 (0.00)	0 (0.00)	0 (0.00)	2 (0.08)	2 (0.02)
**Unsolicited**	28 (1.10)	4 (0.16)	2 (0.08)	15 (0.60)	49 (0.49)

Data are no. (%) of participants.

In the passive AEFI surveillance, 57 AEFI cases following live attenuated mumps vaccine were reported, with an incidence of 19.28/100000 (57/287608). Most AEFI cases were common adverse reactions (66.67%, 38/57); coincidental events and psychogenic reactions only accounted for 19.30% (11/57) and 8.77% (5/57) of cases, respectively. Injection-site pain, myasthenia, injection-site swelling, and fever were the most frequently reported common adverse reactions. No vaccine quality events or program errors were reported. The incidence of AEFI symptoms after vaccination was provided in the S2 Table.

## Discussion

Mumps has become a public health problem due to the global resurgence among adolescents in recent years. In order to ensure long-lasting immunity against mumps, the WHO has recommended the introduction of a second dose of mumps-containing vaccine. In China, MMR vaccine has been included in immunization program since 2008 and almost all children receive by now a single dose of MMR at 18 months of age. The live attenuated mumps vaccine (S79 strain) produced by Sinovac (Dalian) Vaccine Technology Co., Ltd. vaccine was approved for marketing in China in 2016 and an extensive post-marketing safety assessment in the context of large-scale applications was necessary. In this study, this vaccine was tested as a booster immunization in China: a combination of active surveillance and passive surveillance were used to evaluate the safety of post-vaccination and a good safety profile was demonstrated.

Our study showed that the overall incidence of adverse reactions was 2.13%, which was significantly lower than the 18.5% in a previous booster immunization trial [24]. This may be related to the fact that the subjects of our study were junior high school students no more than 14 years old, while the subjects of the booster immunization trial were conducted in children aged 3–6 years old. Additionally, this study was conducted in four research sites, but the incidence of adverse reactions was significantly different among the four sites. One possible reason was that this study was carried out by relying on the widely distributed community health service centers and vaccination clinics, but the intensity of surveillance may vary across study sites, leading to differences in observation results. Another possible reason may be related to local environmental and individual variations.

In accordance with the present findings, most of the solicited local and systemic adverse reactions caused by the mumps vaccine were mild or moderate in intensity, and the participants recovered without any complications. A few grade 3 adverse reactions were reported, the incidence of which was less than 1%. These data, together with the fact that no serious adverse events were reported throughout the trial, indicated a favorable safety profile for the investigational vaccine in this trial. Injection-site pain was the most common local symptom, which was consistent with other published clinical studies [24,25]. In general, fever was the most frequently reported systemic adverse reaction, whereas in the present study, nausea was the main systemic adverse reaction, followed by fever. Collectively, the symptoms of adverse reactions in this trial were basically consistent with previous researches.

A previous clinical trial indicated that adverse reactions to the live attenuated mumps-containing vaccine mainly occurred within 3 days after vaccination [26]. However, in the present study, almost 80% of adverse reactions developed within 1 day after administration. A potential explanation for this was that the awareness of parents or legal guardians decreased over time. Additionally, some mild adverse reactions may have occurred but were not reported after the first day. This phenomenon also reminds doctors and parents about the importance of closely observing the physical manifestations of the recipients within 1 day post-inoculation, especially during the first 30 minutes after vaccination, so as to detect and deal with adverse reactions in time.

In the current study, the incidence of AEFI in passive surveillance was 19.28/100000, which was significantly lower than that in active surveillance. Similar phenomena were also observed in post-marketing studies of other vaccines [27]. Although the passive vaccine safety surveillance system has the advantages of being cost-effective, convenient and broad-reaching, it has low sensitivity and cannot prove causality alone [27]. This is due to under-reporting and reporting bias of adverse events, which is an inherent limitation of a passive reporting systems [22].

One of the strengths of this study was that the safety data were collected by a combination of active surveillance and passive surveillance. We knew that safety monitoring in post-licensure surveillance mainly relies on passive reporting systems and epidemiological research, while our study could perform a comprehensive safety evaluation. However, this study also has some limitations. First, previous data on mumps vaccination and infection were not collected. There has been previous evidence that the incidence of adverse events after MMR vaccination in adults may be related to whether or not subjects had received MMR in childhood [28]. Another potential limitation of this trial is that it was an open-label design, which could theoretically bias the reporting of adverse reactions and safety assessments. Finally, given that some adverse reactions may be related to underlying diseases or conditions, to accidental events shortly after vaccination, or to drugs or other vaccines given concurrently, it was difficult to obtain accurate safety information.

To conclude, this phase 4, open-label, observational trial showed that the live attenuated mumps vaccine produced by Sinovac (Dalian) was generally safe, well-tolerated and suitable for large-scale inoculation.

## Supporting information

S1 Table Summary of adverse reaction symptoms by severity. (DOCX)

S2 Table Incidence of AEFI symptoms after vaccination. (DOCX)

S1 Data (ZIP)
