# Supplementary material for: Safety analysis of a live attenuated mumps vaccine in healthy adolescents in China: A phase 4, observational, open-label trial
Source: PLoS One. 2023 Sep 21;18(9):e0291730. doi: 10.1371/journal.pone.0291730 (PMC10513284; doi:10.1371/journal.pone.0291730)
Supplement: S1 Table — (DOCX) [file pone.0291730.s001.docx]

**Table S1: Summary of adverse reaction symptoms by severity.**

| **Reaction type and severity** | **Baoji City**  **(n = 2557)** | **Hanzhong City**  **(n = 2500)** | **Xianyang City**  **(n = 2500)** | **Yan'an City**  **(n = 2500)** | **Total**  **(N = 10057)** |
| --- | --- | --- | --- | --- | --- |
| **Solicited** | 67 (2.62) | 19 (0.76) | 37 (1.48) | 51 (2.04) | 174 (1.73) |
| **Local** | 57 (2.23) | 8 (0.32) | 35 (1.40) | 37 (1.48) | 137 (1.36) |
| Rash | 3 (0.12) | 0 (0.00) | 0 (0.00) | 7 (0.28) | 10 (0.10) |
| Grade 1 | 0 (0.00) | 0 (0.00) | 0 (0.00) | 5 (0.20) | 5 (0.05) |
| Grade 2 | 3 (0.12) | 0 (0.00) | 0 (0.00) | 1 (0.04) | 4 (0.04) |
| Grade 3 | 0 (0.00) | 0 (0.00) | 0 (0.00) | 1 (0.04) | 1 (0.01) |
| Pain | 30 (1.17) | 5 (0.20) | 19 (0.76) | 17 (0.68) | 71 (0.71) |
| Grade 1 | 26 (1.02) | 5 (0.20) | 11 (0.44) | 14 (0.56) | 56 (0.56) |
| Grade 2 | 4 (0.16) | 0 (0.00) | 8 (0.32) | 2 (0.08) | 14 (0.14) |
| Grade 3 | 0 (0.00) | 0 (0.00) | 0 (0.00) | 1 (0.04) | 1 (0.01) |
| Swelling | 3 (0.12) | 0 (0.00) | 4 (0.16) | 10 (0.40) | 17 (0.17) |
| Grade 1 | 3 (0.12) | 0 (0.00) | 4 (0.16) | 8 (0.32) | 15 (0.15) |
| Grade 2 | 0 (0.00) | 0 (0.00) | 0 (0.00) | 1 (0.04) | 1 (0.04) |
| Grade 3 | 0 (0.00) | 0 (0.00) | 0 (0.00) | 1 (0.04) | 1 (0.01) |
| Pruritus | 7 (0.27) | 0 (0.00) | 6 (0.24) | 4 (0.16) | 17 (0.17) |
| Grade 1 | 7 (0.27) | 0 (0.00) | 6 (0.24) | 2 (0.08) | 15 (0.15) |
| Grade 2 | 0 (0.00) | 0 (0.00) | 0 (0.00) | 2 (0.08) | 2 (0.02) |
| Redness | 14 (0.55) | 2 (0.08) | 10 (0.40) | 3 (0.12) | 29 (0.29) |
| Grade 1 | 14 (0.55) | 2 (0.08) | 10 (0.40) | 2 (0.08) | 28 (0.28) |
| Grade 3 | 0 (0.00) | 0 (0.00) | 0 (0.00) | 1 (0.04) | 1 (0.01) |
| Induration | 0 (0.00) | 1 (0.04) | 3 (0.12) | 6 (0.24) | 10 (0.10) |
| Grade 1 | 0 (0.00) | 1 (0.04) | 3 (0.12) | 6 (0.24) | 10 (0.10) |
| **Systemic** | 11 (0.43) | 11 (0.44) | 4 (0.16) | 25 (1.00) | 51 (0.51) |
| Nausea | 4 (0.16) | 2 (0.08) | 0 (0.00) | 13 (0.52) | 19 (0.19) |
| Grade 1 | 4 (0.16) | 2 (0.08) | 0 (0.00) | 7 (0.28) | 13 (0.13) |
| Grade 2 | 0 (0.00) | 0 (0.00) | 0 (0.00) | 6 (0.24) | 6 (0.06) |
| Vomiting | 2 (0.08) | 1 (0.04) | 0 (0.00) | 3 (0.12) | 6 (0.06) |
| Grade 1 | 1 (0.04) | 1 (0.04) | 0 (0.00) | 2 (0.08) | 4 (0.04) |
| Grade 2 | 1 (0.04) | 0 (0.00) | 0 (0.00) | 1 (0.04) | 2 (0.02) |
| Diarrhea | 0 (0.00) | 1 (0.04) | 0 (0.00) | 1 (0.04) | 2 (0.02) |
| Grade 1 | 0 (0.00) | 1 (0.04) | 0 (0.00) | 0 (0.00) | 1 (0.01) |
| Grade 2 | 0 (0.00) | 0 (0.00) | 0 (0.00) | 1 (0.04) | 1 (0.01) |
| Fever | 4 (0.16) | 6 (0.24) | 2 (0.08) | 4 (0.16) | 16 (0.16) |
| Grade 1 | 2 (0.08) | 5 (0.20) | 0 (0.00) | 2 (0.08) | 9 (0.09) |
| Grade 2 | 2 (0.08) | 1 (0.04) | 2 (0.08) | 2 (0.08) | 7 (0.07) |
| Fatigue | 0 (0.00) | 0 (0.00) | 0 (0.00) | 2 (0.08) | 2 (0.02) |
| Grade 2 | 0 (0.00) | 0 (0.00) | 0 (0.00) | 1 (0.04) | 1 (0.01) |
| Grade 3 | 0 (0.00) | 0 (0.00) | 0 (0.00) | 1 (0.04) | 1 (0.01) |
| Skin and mucosa  abnormality | 1 (0.04) | 0 (0.00) | 0 (0.00) | 1 (0.04) | 2 (0.02) |
| Grade 2 | 1 (0.04) | 0 (0.00) | 0 (0.00) | 1 (0.04) | 2 (0.02) |
| Cough | 3 (0.12) | 0 (0.00) | 0 (0.00) | 2 (0.08) | 5 (0.05) |
| Grade 1 | 3 (0.12) | 0 (0.00) | 0 (0.00) | 1 (0.04) | 4 (0.04) |
| Grade 2 | 0 (0.00) | 0 (0.00) | 0 (0.00) | 1 (0.04) | 1 (0.01) |
| Headache | 2 (0.08) | 2 (0.08) | 2 (0.08) | 2 (0.08) | 8 (0.08) |
| Grade 1 | 2 (0.08) | 2 (0.08) | 2 (0.08) | 2 (0.08) | 8 (0.08) |
| Decreased appetite | 0 (0.00) | 1 (0.04) | 0 (0.00) | 0 (0.00) | 1 (0.01) |
| Grade 1 | 0 (0.00) | 1 (0.04) | 0 (0.00) | 0 (0.00) | 1 (0.01) |
| Muscle pain | 0 (0.00) | 0 (0.00) | 0 (0.00) | 2 (0.08) | 2 (0.02) |
| Grade 2 | 0 (0.00) | 0 (0.00) | 0 (0.00) | 1 (0.04) | 1 (0.01) |
| Grade 3 | 0 (0.00) | 0 (0.00) | 0 (0.00) | 1 (0.04) | 1 (0.01) |
| **Unsolicited** | 28 (1.10) | 4 (0.16) | 2 (0.08) | 15 (0.60) | 49 (0.49) |
| **Gastrointestinal disorders** | 2 (0.08) | 0 (0.00) | 0 (0.00) | 2 (0.08) | 4 (0.04) |
| Enteritis | 0 (0.00) | 0 (0.00) | 0 (0.00) | 1 (0.04) | 1 (0.01) |
| Grade 2 | 0 (0.00) | 0 (0.00) | 0 (0.00) | 1 (0.04) | 1 (0.01) |
| Abdominal pain | 1 (0.04) | 0 (0.00) | 0 (0.00) | 1 (0.04) | 2 (0.02) |
| Grade 2 | 1 (0.04) | 0 (0.00) | 0 (0.00) | 1 (0.04) | 2 (0.02) |
| Epigastric pain | 1 (0.04) | 0 (0.00) | 0 (0.00) | 0 (0.00) | 1 (0.01) |
| Grade 1 | 1 (0.04) | 0 (0.00) | 0 (0.00) | 0 (0.00) | 1 (0.01) |
| **General disorders and administration**  **site conditions** | 0 (0.00) | 0 (0.00) | 0 (0.00) | 2 (0.08) | 2 (0.02) |
| Fever | 0 (0.00) | 0 (0.00) | 0 (0.00) | 1 (0.04) | 1 (0.01) |
| Grade 2 | 0 (0.00) | 0 (0.00) | 0 (0.00) | 1 (0.04) | 1 (0.01) |
| Chest discomfort | 0 (0.00) | 0 (0.00) | 0 (0.00) | 1 (0.04) | 1 (0.01) |
| Grade 3 | 0 (0.00) | 0 (0.00) | 0 (0.00) | 1 (0.04) | 1 (0.01) |
| **Skin and subcutaneous tissue disorders** | 0 (0.00) | 1 (0.04) | 2 (0.08) | 0 (0.00) | 3 (0.03) |
| Urticaria | 0 (0.00) | 0 (0.00) | 1 (0.04) | 0 (0.00) | 1 (0.01) |
| Grade 2 | 0 (0.00) | 0 (0.00) | 1 (0.04) | 0 (0.00) | 1 (0.01) |
| Rash | 0 (0.00) | 1 (0.04) | 0 (0.00) | 0 (0.00) | 1 (0.01) |
| Grade 3 | 0 (0.00) | 1 (0.04) | 0 (0.00) | 0 (0.00) | 1 (0.01) |
| Allergic dermatitis | 0 (0.00) | 0 (0.00) | 1 (0.04) | 0 (0.00) | 1 (0.01) |
| Grade 1 | 0 (0.00) | 0 (0.00) | 1 (0.04) | 0 (0.00) | 1 (0.01) |
| **Respiratory, thoracic and mediastinal**  **disorders** | 0 (0.00) | 0 (0.00) | 0 (0.00) | 1 (0.04) | 1 (0.01) |
| Dyspnea | 0 (0.00) | 0 (0.00) | 0 (0.00) | 1 (0.04) | 1 (0.01) |
| Grade 3 | 0 (0.00) | 0 (0.00) | 0 (0.00) | 1 (0.04) | 1 (0.01) |
| **Nervous system disorders** | 1 (0.04) | 2 (0.08) | 0 (0.00) | 11 (0.44) | 14 (0.14) |
| Dizziness | 1 (0.04) | 2 (0.08) | 0 (0.00) | 10 (0.40) | 13 (0.13) |
| Grade 1 | 1 (0.04) | 2 (0.08) | 0 (0.00) | 0 (0.00) | 3 (0.03) |
| Grade 2 | 0 (0.00) | 0 (0.00) | 0 (0.00) | 9 (0.36) | 9 (0.09) |
| Grade 3 | 0 (0.00) | 0 (0.00) | 0 (0.00) | 1 (0.04) | 1 (0.01) |
| Hypoesthesia | 0 (0.00) | 0 (0.00) | 0 (0.00) | 1 (0.04) | 1 (0.01) |
| Grade 3 | 0 (0.00) | 0 (0.00) | 0 (0.00) | 1 (0.04) | 1 (0.01) |
| **Musculoskeletal and connective**  **tissue disorders** | 4 (0.16) | 0 (0.00) | 0 (0.00) | 0 (0.00) | 4 (0.04) |
| Back pain | 1 (0.04) | 0 (0.00) | 0 (0.00) | 0 (0.00) | 1 (0.01) |
| Grade 2 | 1 (0.04) | 0 (0.00) | 0 (0.00) | 0 (0.00) | 1 (0.01) |
| Joint pain | 2 (0.08) | 0 (0.00) | 0 (0.00) | 0 (0.00) | 2 (0.02) |
| Grade 1 | 2 (0.08) | 0 (0.00) | 0 (0.00) | 0 (0.00) | 2 (0.02) |
| Myalgia | 1 (0.04) | 0 (0.00) | 0 (0.00) | 0 (0.00) | 1 (0.01) |
| Grade 2 | 1 (0.04) | 0 (0.00) | 0 (0.00) | 0 (0.00) | 1 (0.01) |
| **Infections and infestations** | 19 (0.74) | 1 (0.04) | 0 (0.00) | 2 (0.08) | 22 (0.22) |
| Pharyngitis | 0 (0.00) | 1 (0.04) | 0 (0.00) | 0 (0.00) | 1 (0.01) |
| Grade 1 | 0 (0.00) | 1 (0.04) | 0 (0.00) | 0 (0.00) | 1 (0.01) |
| Tonsillitis | 1 (0.04) | 0 (0.00) | 0 (0.00) | 0 (0.00) | 1 (0.01) |
| Grade 2 | 1 (0.04) | 0 (0.00) | 0 (0.00) | 0 (0.00) | 1 (0.01) |
| Upper respiratory infection | 18 (0.70) | 0 (0.00) | 0 (0.00) | 2 (0.08) | 20 (0.20) |
| Grade 1 | 9 (0.35) | 0 (0.00) | 0 (0.00) | 0 (0.00) | 9 (0.09) |
| Grade 2 | 9 (0.35) | 0 (0.00) | 0 (0.00) | 2 (0.08) | 11 (0.11) |
| **Vascular and lymphatic diseases** | 0 (0.00) | 0 (0.00) | 0 (0.00) | 1 (0.04) | 1 (0.01) |
| Flush | 0 (0.00) | 0 (0.00) | 0 (0.00) | 1 (0.04) | 1 (0.01) |
| Grade 2 | 0 (0.00) | 0 (0.00) | 0 (0.00) | 1 (0.04) | 1 (0.01) |
| **Blood and lymphatic system disorders** | 2 (0.08) | 0 (0.00) | 0 (0.00) | 0 (0.00) | 2 (0.02) |
| Lymphadenitis | 2 (0.08) | 0 (0.00) | 0 (0.00) | 0 (0.00) | 2 (0.02) |
| Grade 2 | 2 (0.08) | 0 (0.00) | 0 (0.00) | 0 (0.00) | 2 (0.02) |
| **Immune system disorders** | 1 (0.04) | 0 (0.00) | 0 (0.00) | 0 (0.00) | 1 (0.01) |
| Hypersensitivity | 1 (0.04) | 0 (0.00) | 0 (0.00) | 0 (0.00) | 1 (0.01) |
| Grade 2 | 1 (0.04) | 0 (0.00) | 0 (0.00) | 0 (0.00) | 1 (0.01) |

Data are no. (%) of participants.
