# Supplementary material for: Safety analysis of a live attenuated mumps vaccine in healthy adolescents in China: A phase 4, observational, open-label trial
Source: PLoS One. 2023 Sep 21;18(9):e0291730. doi: 10.1371/journal.pone.0291730 (PMC10513284; doi:10.1371/journal.pone.0291730)
Supplement: S2 Table — (DOCX) [file pone.0291730.s002.docx]

**Table S2: Incidence of AEFI symptoms after vaccination.**

| **AEFI symptoms** |  | **Total (N=287608)** | |
| --- | --- | --- | --- |
|  |  | **AEFI cases** | **IR (per 100000 doses)** |
| **Common adverse reaction** |  | 38 | 13.21 |
| Injection-site rash |  | 1 | 0.35 |
| Injection-site pain |  | 12 | 4.17 |
| Injection-site pruritus |  | 2 | 0.70 |
| Injection-site swelling |  | 5 | 1.74 |
| Injection-site erythema |  | 1 | 0.35 |
| Injection-site induration |  | 2 | 0.70 |
| Fever |  | 5 | 1.74 |
| Peripheral swelling |  | 1 | 0.35 |
| Chest pain |  | 1 | 0.35 |
| Rash |  | 1 | 0.35 |
| Cough |  | 1 | 0.35 |
| Dizziness |  | 1 | 0.35 |
| Joint pain |  | 2 | 0.70 |
| Myasthenia |  | 8 | 2.78 |
| Limb pain |  | 3 | 1.04 |
| Skeletal muscle pain |  | 1 | 0.35 |
| Gastrointestinal cold |  | 1 | 0.35 |
| Flush |  | 2 | 0.70 |
| **Rare adverse reaction** |  | 3 | 1.04 |
| Abdominal pain |  | 1 | 0.35 |
| Hypoesthesia |  | 1 | 0.35 |
| Allergic dermatitis |  | 1 | 0.35 |
| **Coincidental event** |  | 11 | 3.83 |
| Chest discomfort |  | 2 | 0.70 |
| Enteritis |  | 1 | 0.35 |
| Dyspnea |  | 2 | 0.70 |
| Tonsillitis |  | 1 | 0.35 |
| Gastroenteritis |  | 2 | 0.70 |
| Infection upper respiratory |  | 5 | 1.74 |
| **Psychogenic reaction** |  | 5 | 1.74 |
| Barriers to change |  | 5 | 1.74 |
| **Total** |  | 57 | 19.82 |
