# Supplementary material for: Safety analysis of a live attenuated mumps vaccine in healthy adolescents in China: A phase 4, observational, open-label trial
Source: PLoS One. 2023 Sep 21;18(9):e0291730. doi: 10.1371/journal.pone.0291730 (PMC10513284; doi:10.1371/journal.pone.0291730)
Supplement: S1 Data — (ZIP) [file pone.0291730.s003.zip › S1_Data.docx]

**Safety analysis of a live attenuated mumps vaccine in healthy adolescents in China: A phase 4, observational, open-label trial**

Protocol No.: PRO-MUMPS-4007

Contents

[1. Abbreviations and statistics used below 4](#_Toc145065068)

[2. Purpose 4](#_Toc145065069)

[3. Design 4](#_Toc145065070)

[3.1. Overall Design 4](#_Toc145065071)

[3.2. Sample Size 4](#_Toc145065072)

[3.3. Coding for the Participants 5](#_Toc145065073)

[4. Endpoint 5](#_Toc145065074)

[5. Analysis Set 5](#_Toc145065075)

[5.1. Safety Set (SS) 5](#_Toc145065076)

[6.1. Statistical Description 5](#_Toc145065077)

[6.2. Analysis indicators 5](#_Toc145065078)

[6.3. Group Analysis 5](#_Toc145065079)

[6.4. Demographics and the Baseline Characteristics 5](#_Toc145065080)

[6.5. Statistical Inference 6](#_Toc145065081)

[7. Notes on the Plan 6](#_Toc145065082)

[8. Statistical analysis tables 7](#_Toc145065083)

[8.1. Study population 7](#_Toc145065084)

[8.1.1. Study execution 7](#_Toc145065085)

[8.1.2. Demographic characteristics 7](#_Toc145065086)

[8.2. Safety Evaluation 8](#_Toc145065087)

[8.2.1. Adverse Events 8](#_Toc145065088)

[8.2.2. Analysis of adverse event following immunization(AEFI) 24](#_Toc145065089)

[**Version History** 28](#_Toc145065090)

# Abbreviations and statistics used below

| CFDA | China Food and Drug Administration |
| --- | --- |
| NMPA | National Medical Products Administration |
| CDC | Center for Disease Control and Prevention |
| GMT | Geometric Mean Titer |
| IEC | Independent Ethics Committee |
| PI | Principal Investigator |
| SOP | Standard Operation Procedure |
| MMR | Measles, Mumps and Rubella Vaccine |
| MV | Mumps Vaccine |
| AE | Adverse Event |
| AEFI | Adverse Events Following Immunization |
| SS | Safety Set |

# Purpose

To evaluate the safety of MV after large-scale application, accumulate safety data for the application of MV, and provide a scientific basis for the development of vaccine immunization prevention strategies. Specific indicators include:

- The incidence of local and systemic adverse reactions within 30 minutes after vaccination;
- The incidence of local and systemic adverse reactions at 0~14 days after vaccination;
- The incidence of local and systemic adverse reactions at 0~30 days after vaccination.

# Design

## Overall Design

Safety observation is conducted using an open-label design, combining active monitoring and passive monitoring. Several vaccination units in the project area are selected as the study sites. The study population consists of first-year students from middle school, and active monitoring is conducted by face-to-face interviews or telephone follow-ups at 30 minutes, 14 days, and 30 days after each dose of vaccination to collect adverse reactions/events. Passive monitoring is conducted to collect adverse reactions related to MV through the database of "Suspected Adverse Reaction Monitoring System for Preventive Vaccination".

## Sample Size

The post-market safety observation study aims to identify the rare adverse reactions that may not be detected in pre-market studies due to the small sample size. Therefore, according to the requirements of vaccine research and development, as well as the original CFDA drug registration approval: "Active safety monitoring should be strengthened during marketing use", The predetermined sample size for active monitoring is set at 10,000 doses.

## Coding for the Participants

The coding principle is SX + 2 letters + 6 digits, where SX represents the project province, 2 letters represent the abbreviation of the district or county. The first 2 digits of the 6-digit number represent the vaccination unit that determined by the district or county CDC (Center for Disease Control and Prevention), using consecutive numbering from 01 to 99. The last 4 digits represent the observed subjects vaccinated at that vaccination unit, using consecutive numbering from 0001 to 9999.

# Endpoint

The incidence of adverse reactions at 0~14 days and 0~30 days after vaccination.

# Analysis Set

## Safety Set (SS)

All subjects who have completed vaccination and have safety assessment information are included.

1. **Statistical Method**

## Statistical Description

Quantitative indicators are presented as mean ± standard deviation, median, maximum value, and minimum value. Qualitative or ordinal indicators are presented as frequency distribution tables.

## Analysis indicators

The incidence rates of systemic and local adverse reactions/events within 30 minutes after vaccination, the incidence rates of systemic and local adverse reactions/events at 0~14 days after vaccination, and the incidence rates of systemic and local adverse reactions/events at 0~30 days after vaccination.

## Group Analysis

Describing the incidence rates of adverse reactions/events in different regions and genders, and comparing whether there are statistically significant differences between the groups.

## Demographics and the Baseline Characteristics

Descriptive statistical analysis is conducted on the demographic characteristics of each group of participants, including gender, age, etc. Based on the distribution characteristics of the variables, analysis of variance (ANOVA) or Wilcoxon rank-sum test is used to statistically compare the differences between groups for variables such as age. Chi-square test or Fisher's exact test is used to statistically compare differences between groups for variables such as gender.

## Statistical Inference

Appropriate statistical analysis methods are used for within-group or between-group comparisons based on different applicable conditions. All statistical tests provide the test statistic and the corresponding p-value, and when using the exact probability method (Fisher's method), the p-value is directly provided. All statistical tests use two-tailed tests, and a p-value less than or equal to 0.05 is considered statistically significant for the tested difference (unless otherwise specified).

# Notes on the Plan

This analysis plan was drafted based on the relevant description in the study protocol and defined the indicators of safety evaluation. According to the basic characteristics of each index in the protocol, combined with the specific requirements of this study, the specific statistical analysis method of the relevant evaluation indicators is proposed. Considering that there may be some unexpected changes in the final data distribution form of clinical trials, the statistical analysis method may be slightly adjusted, and the presentation of the corresponding statistical analysis results may also change to a certain extent.

# Statistical analysis tables

## Study population

### Study execution

Table 8.1.1.1 Safety analysis

|  | **Baoji City** | **Hanzhong City** | **Xianyang City** | **Yan’an City** | **Total** |
| --- | --- | --- | --- | --- | --- |
| Vaccinated | 2557 | 2500 | 2500 | 2500 | 10057 |
| Safety analysis population (%) | 2557(100.00) | 2500(100.00) | 2500(100.00) | 2500(100.00) | 10057(100.00) |

### Demographic characteristics

Table 8.1.2.1 Demographic characteristics and physical examination in different regions（SS）

|  | **Baoji City(%)** | **Hanzhong City(%)** | **Xianyang City(%)** | **Yan’an City(%)** | **Total** | **Method** | **Statistics** | **P** |
| --- | --- | --- | --- | --- | --- | --- | --- | --- |
| Age |  |  |  |  |  |  |  |  |
| N (Missing) | 2557(0) | 2500(0) | 2500(0) | 2500(0) | 10057(0) | ANOVA/ Wilcoxon rank-sum test | 322.254 | <0.0001 |
| Mean (SD) | 11.90(0.72) | 12.13(0.71) | 12.23(0.71) | 12.22(0.78) | 12.12(0.74) |  |  |  |
| Median | 12 | 12 | 12 | 12 | 12 |  |  |  |
| Min, Max | 10, 19 | 10, 14 | 10, 15 | 10, 18 | 10, 19 |  |  |  |
| Sex |  |  |  |  |  |  |  |  |
| Male n(%) | 1378(53.89) | 1299(51.96) | 1355(54.20) | 1363(54.52) | 5395(53.64) | CHI-SQUARE/ Fisher’s Exact Test | 3.996 | 0.2619 |
| Female n(%) | 1179(46.11) | 1201(48.04) | 1145(45.80) | 1137(45.48) | 4662(46.36) |  |  |  |
| N (Missing) | 2557(0) | 2500(0) | 2500(0) | 2500(0) | 10057(0) |  |  |  |

## Safety Evaluation

### Adverse Events

#### Overall adverse events

Table 8.2.1.1.1 Summary table of adverse events（SS）

|  | **Baoji City**  **(N=2557)** | | |  | **Hanzhong City**  **(N=2500)** | | |  | **Xianyang City**  **(N=2500)** | | | |  | | **Yan’an City**  **(N=2500)** | | | |  | | **Total**  **(N=10057)** | | | |  | | **Fisher P** | |  |
| --- | --- | --- | --- | --- | --- | --- | --- | --- | --- | --- | --- | --- | --- | --- | --- | --- | --- | --- | --- | --- | --- | --- | --- | --- | --- | --- | --- | --- | --- |
|  | **No. of events** | **No. of subjects** | **Incidence rate(%)** |  | **No. of events** | **No. of subjects** | **Incidence rate(%)** |  | | **No. of events** | **No. of subjects** | **Incidence rate(%)** | |  | | **No. of events** | **No. of subjects** | **Incidence rate(%)** | |  | | **No. of events** | **No. of subjects** | **Incidence rate(%)** | |  | |  | |
| Overall adverse events | 350 | 308 | 12.05 |  | 222 | 195 | 7.80 |  | | 69 | 57 | 2.28 | |  | | 422 | 292 | 11.68 | |  | | 1063 | 852 | 8.47 | |  | | <0.0001 | |
| Vaccination-related | 102 | 94 | 3.68 |  | 25 | 21 | 0.84 |  | | 49 | 38 | 1.52 | |  | | 105 | 61 | 2.44 | |  | | 281 | 214 | 2.13 | |  | | <0.0001 | |
| Vaccination-unrelated | 248 | 219 | 8.56 |  | 197 | 176 | 7.04 |  | | 20 | 19 | 0.76 | |  | | 317 | 249 | 9.96 | |  | | 782 | 663 | 6.59 | |  | | <0.0001 | |

Note：(1)Vaccination-related refers to the relationship between adverse events and vaccines as ’possibly related’, ’highly likely related’ and ’definitely related’.

(2)Vaccination-related refers to the relationship between adverse events and vaccines as ’possibly unrelated’ and ’definitely unrelated’.

Table 8.2.1.1.2 Incidence and frequency of adverse events (by solicited and unsolicited)

|  | **Baoji City**  **(N=2557)** | | |  | **Hanzhong City**  **(N=2500)** | | | |  | **Xianyang City**  **(N=2500)** | | |  | **Yan’an City**  **(N=2500)** | | | |  | | **Total**  **(N=10057)** | | | |  | | **Fisher P** | |  |
| --- | --- | --- | --- | --- | --- | --- | --- | --- | --- | --- | --- | --- | --- | --- | --- | --- | --- | --- | --- | --- | --- | --- | --- | --- | --- | --- | --- | --- |
| **Adverse event term** | **No. of events** | **No. of subjects** | **Incidence rate(%)** |  | | **No. of events** | **No. of subjects** | **Incidence rate(%)** |  | **No. of events** | **No. of subjects** | **Incidence rate(%)** |  | | **No. of events** | **No. of subjects** | **Incidence rate(%)** | |  | | **No. of events** | **No. of subjects** | **Incidence rate(%)** | |  | |  | |
| **Solicited** | 98 | 90 | 3.52 |  | | 68 | 50 | 2.00 |  | 53 | 42 | 1.68 |  | | 185 | 131 | 5.24 | |  | | 404 | 313 | 3.11 | |  | | <0.0001 | |
| **Local adverse event** | 57 | 57 | 2.23 |  | | 8 | 8 | 0.32 |  | 43 | 35 | 1.40 |  | | 52 | 37 | 1.48 | |  | | 160 | 137 | 1.36 | |  | | <0.0001 | |
| General disorders and administration site conditions | 57 | 57 | 2.23 |  | | 8 | 8 | 0.32 |  | 43 | 35 | 1.40 |  | | 52 | 37 | 1.48 | |  | | 160 | 137 | 1.36 | |  | | <0.0001 | |
| Vaccination site rash | 3 | 3 | 0.12 |  | | 0 | 0 | 0.00 |  | 0 | 0 | 0.00 |  | | 10 | 7 | 0.28 | |  | | 13 | 10 | 0.10 | |  | | 0.0026 | |
| Vaccination site pain | 30 | 30 | 1.17 |  | | 5 | 5 | 0.20 |  | 19 | 19 | 0.76 |  | | 17 | 17 | 0.68 | |  | | 71 | 71 | 0.71 | |  | | 0.0003 | |
| Vaccination site swelling | 3 | 3 | 0.12 |  | | 0 | 0 | 0.00 |  | 4 | 4 | 0.16 |  | | 11 | 10 | 0.40 | |  | | 18 | 17 | 0.17 | |  | | 0.0033 | |
| Vaccination site pruritus | 7 | 7 | 0.27 |  | | 0 | 0 | 0.00 |  | 7 | 6 | 0.24 |  | | 4 | 4 | 0.16 | |  | | 18 | 17 | 0.17 | |  | | 0.0379 | |
| Injection site erythema | 14 | 14 | 0.55 |  | | 2 | 2 | 0.08 |  | 10 | 10 | 0.40 |  | | 3 | 3 | 0.12 | |  | | 29 | 29 | 0.29 | |  | | 0.0034 | |
| Injection site induration | 0 | 0 | 0.00 |  | | 1 | 1 | 0.04 |  | 3 | 3 | 0.12 |  | | 7 | 6 | 0.24 | |  | | 11 | 10 | 0.10 | |  | | 0.0216 | |
| **Systemic adverse event** | 41 | 34 | 1.33 |  | | 60 | 42 | 1.68 |  | 10 | 9 | 0.36 |  | | 133 | 109 | 4.36 | |  | | 244 | 194 | 1.93 | |  | | <0.0001 | |
| Gastrointestinal Disorders | 9 | 7 | 0.27 |  | | 13 | 9 | 0.36 |  | 0 | 0 | 0.00 |  | | 39 | 27 | 1.08 | |  | | 61 | 43 | 0.43 | |  | | <0.0001 | |
| Nausea | 5 | 5 | 0.20 |  | | 5 | 4 | 0.16 |  | 0 | 0 | 0.00 |  | | 21 | 15 | 0.60 | |  | | 31 | 24 | 0.24 | |  | | <0.0001 | |
| Vomiting | 4 | 4 | 0.16 |  | | 5 | 4 | 0.16 |  | 0 | 0 | 0.00 |  | | 8 | 8 | 0.32 | |  | | 17 | 16 | 0.16 | |  | | 0.0259 | |
| Diarrhoea | 0 | 0 | 0.00 |  | | 3 | 3 | 0.12 |  | 0 | 0 | 0.00 |  | | 10 | 9 | 0.36 | |  | | 13 | 12 | 0.12 | |  | | 0.0002 | |
| General disorders and administration site conditions | 9 | 9 | 0.35 |  | | 13 | 13 | 0.52 |  | 2 | 2 | 0.08 |  | | 24 | 23 | 0.92 | |  | | 48 | 47 | 0.47 | |  | | <0.0001 | |
| Pyrexia | 7 | 7 | 0.27 |  | | 6 | 6 | 0.24 |  | 2 | 2 | 0.08 |  | | 16 | 16 | 0.64 | |  | | 31 | 31 | 0.31 | |  | | 0.0046 | |
| Asthenia | 2 | 2 | 0.08 |  | | 7 | 7 | 0.28 |  | 0 | 0 | 0.00 |  | | 8 | 7 | 0.28 | |  | | 17 | 16 | 0.16 | |  | | 0.0086 | |
| Skin and subcutaneous tissue disorders | 2 | 2 | 0.08 |  | | 0 | 0 | 0.00 |  | 0 | 0 | 0.00 |  | | 1 | 1 | 0.04 | |  | | 3 | 3 | 0.03 | |  | | 0.6249 | |
| Mucocutaneous rash | 2 | 2 | 0.08 |  | | 0 | 0 | 0.00 |  | 0 | 0 | 0.00 |  | | 1 | 1 | 0.04 | |  | | 3 | 3 | 0.03 | |  | | 0.6249 | |
| Respiratory, thoracic and mediastinal disorders | 15 | 15 | 0.59 |  | | 22 | 22 | 0.88 |  | 4 | 4 | 0.16 |  | | 59 | 59 | 2.36 | |  | | 100 | 100 | 0.99 | |  | | <0.0001 | |
| Cough | 15 | 15 | 0.59 |  | | 22 | 22 | 0.88 |  | 4 | 4 | 0.16 |  | | 59 | 59 | 2.36 | |  | | 100 | 100 | 0.99 | |  | | <0.0001 | |
| Nervous system disorders | 6 | 6 | 0.23 |  | | 10 | 10 | 0.40 |  | 4 | 4 | 0.16 |  | | 7 | 7 | 0.28 | |  | | 27 | 27 | 0.27 | |  | | 0.4388 | |
| Headache | 6 | 6 | 0.23 |  | | 10 | 10 | 0.40 |  | 4 | 4 | 0.16 |  | | 7 | 7 | 0.28 | |  | | 27 | 27 | 0.27 | |  | | 0.4388 | |
| Metabolism and nutrition disorders | 0 | 0 | 0.00 |  | | 1 | 1 | 0.04 |  | 0 | 0 | 0.00 |  | | 0 | 0 | 0.00 | |  | | 1 | 1 | 0.01 | |  | | 0.7457 | |
| Decreased appetite | 0 | 0 | 0.00 |  | | 1 | 1 | 0.04 |  | 0 | 0 | 0.00 |  | | 0 | 0 | 0.00 | |  | | 1 | 1 | 0.01 | |  | | 0.7457 | |
| Musculoskeletal and connective tissue disorders | 0 | 0 | 0.00 |  | | 1 | 1 | 0.04 |  | 0 | 0 | 0.00 |  | | 3 | 3 | 0.12 | |  | | 4 | 4 | 0.04 | |  | | 0.1072 | |
| Myalgia | 0 | 0 | 0.00 |  | | 1 | 1 | 0.04 |  | 0 | 0 | 0.00 |  | | 3 | 3 | 0.12 | |  | | 4 | 4 | 0.04 | |  | | 0.1072 | |
| **Unsolicited** | 252 | 231 | 9.03 |  | | 154 | 153 | 6.12 |  | 16 | 16 | 0.64 |  | | 237 | 211 | 8.44 | |  | | 659 | 611 | 6.08 | |  | | <0.0001 | |
| **Systemic adverse event** | 252 | 231 | 9.03 |  | | 154 | 153 | 6.12 |  | 16 | 16 | 0.64 |  | | 237 | 211 | 8.44 | |  | | 659 | 611 | 6.08 | |  | | <0.0001 | |
| Gastrointestinal Disorders | 7 | 7 | 0.27 |  | | 0 | 0 | 0.00 |  | 0 | 0 | 0.00 |  | | 15 | 15 | 0.60 | |  | | 22 | 22 | 0.22 | |  | | <0.0001 | |
| Enteritis | 0 | 0 | 0.00 |  | | 0 | 0 | 0.00 |  | 0 | 0 | 0.00 |  | | 3 | 3 | 0.12 | |  | | 3 | 3 | 0.03 | |  | | 0.0460 | |
| Abdominal pain | 1 | 1 | 0.04 |  | | 0 | 0 | 0.00 |  | 0 | 0 | 0.00 |  | | 6 | 6 | 0.24 | |  | | 7 | 7 | 0.07 | |  | | 0.0040 | |
| Diarrhoea | 1 | 1 | 0.04 |  | | 0 | 0 | 0.00 |  | 0 | 0 | 0.00 |  | | 2 | 2 | 0.08 | |  | | 3 | 3 | 0.03 | |  | | 0.4803 | |
| Mouth ulceration | 2 | 2 | 0.08 |  | | 0 | 0 | 0.00 |  | 0 | 0 | 0.00 |  | | 0 | 0 | 0.00 | |  | | 2 | 2 | 0.02 | |  | | 0.2499 | |
| Vomiting | 0 | 0 | 0.00 |  | | 0 | 0 | 0.00 |  | 0 | 0 | 0.00 |  | | 2 | 2 | 0.08 | |  | | 2 | 2 | 0.02 | |  | | 0.1853 | |
| Gastritis | 1 | 1 | 0.04 |  | | 0 | 0 | 0.00 |  | 0 | 0 | 0.00 |  | | 1 | 1 | 0.04 | |  | | 2 | 2 | 0.02 | |  | | 1.0000 | |
| Dyspepsia | 1 | 1 | 0.04 |  | | 0 | 0 | 0.00 |  | 0 | 0 | 0.00 |  | | 0 | 0 | 0.00 | |  | | 1 | 1 | 0.01 | |  | | 1.0000 | |
| Abdominal pain upper | 1 | 1 | 0.04 |  | | 0 | 0 | 0.00 |  | 0 | 0 | 0.00 |  | | 1 | 1 | 0.04 | |  | | 2 | 2 | 0.02 | |  | | 1.0000 | |
| General disorders and administration site conditions | 0 | 0 | 0.00 |  | | 0 | 0 | 0.00 |  | 0 | 0 | 0.00 |  | | 4 | 4 | 0.16 | |  | | 4 | 4 | 0.04 | |  | | 0.0114 | |
| Pyrexia | 0 | 0 | 0.00 |  | | 0 | 0 | 0.00 |  | 0 | 0 | 0.00 |  | | 3 | 3 | 0.12 | |  | | 3 | 3 | 0.03 | |  | | 0.0460 | |
| Chest discomfort | 0 | 0 | 0.00 |  | | 0 | 0 | 0.00 |  | 0 | 0 | 0.00 |  | | 1 | 1 | 0.04 | |  | | 1 | 1 | 0.01 | |  | | 0.7457 | |
| Skin and subcutaneous tissue disorders | 2 | 2 | 0.08 |  | | 3 | 3 | 0.12 |  | 2 | 2 | 0.08 |  | | 0 | 0 | 0.00 | |  | | 7 | 7 | 0.07 | |  | | 0.4854 | |
| Urticaria | 0 | 0 | 0.00 |  | | 0 | 0 | 0.00 |  | 1 | 1 | 0.04 |  | | 0 | 0 | 0.00 | |  | | 1 | 1 | 0.01 | |  | | 0.7457 | |
| Eczema | 0 | 0 | 0.00 |  | | 1 | 1 | 0.04 |  | 0 | 0 | 0.00 |  | | 0 | 0 | 0.00 | |  | | 1 | 1 | 0.01 | |  | | 0.7457 | |
| Rash | 1 | 1 | 0.04 |  | | 1 | 1 | 0.04 |  | 0 | 0 | 0.00 |  | | 0 | 0 | 0.00 | |  | | 2 | 2 | 0.02 | |  | | 1.0000 | |
| Vitiligo | 1 | 1 | 0.04 |  | | 0 | 0 | 0.00 |  | 0 | 0 | 0.00 |  | | 0 | 0 | 0.00 | |  | | 1 | 1 | 0.01 | |  | | 1.0000 | |
| Dermatitis allergic | 0 | 0 | 0.00 |  | | 1 | 1 | 0.04 |  | 1 | 1 | 0.04 |  | | 0 | 0 | 0.00 | |  | | 2 | 2 | 0.02 | |  | | 0.6207 | |
| Respiratory, thoracic and mediastinal disorders | 1 | 1 | 0.04 |  | | 2 | 2 | 0.08 |  | 0 | 0 | 0.00 |  | | 13 | 12 | 0.48 | |  | | 16 | 15 | 0.15 | |  | | <0.0001 | |
| Cough | 0 | 0 | 0.00 |  | | 0 | 0 | 0.00 |  | 0 | 0 | 0.00 |  | | 5 | 5 | 0.20 | |  | | 5 | 5 | 0.05 | |  | | 0.0028 | |
| Rhinorrhoea | 0 | 0 | 0.00 |  | | 0 | 0 | 0.00 |  | 0 | 0 | 0.00 |  | | 4 | 4 | 0.16 | |  | | 4 | 4 | 0.04 | |  | | 0.0114 | |
| Oropharyngeal pain | 1 | 1 | 0.04 |  | | 0 | 0 | 0.00 |  | 0 | 0 | 0.00 |  | | 3 | 3 | 0.12 | |  | | 4 | 4 | 0.04 | |  | | 0.1540 | |
| Dyspnoea | 0 | 0 | 0.00 |  | | 0 | 0 | 0.00 |  | 0 | 0 | 0.00 |  | | 1 | 1 | 0.04 | |  | | 1 | 1 | 0.01 | |  | | 0.7457 | |
| Rhinitis allergic | 0 | 0 | 0.00 |  | | 2 | 2 | 0.08 |  | 0 | 0 | 0.00 |  | | 0 | 0 | 0.00 | |  | | 2 | 2 | 0.02 | |  | | 0.1853 | |
| Nervous system disorders | 1 | 1 | 0.04 |  | | 3 | 3 | 0.12 |  | 1 | 1 | 0.04 |  | | 20 | 14 | 0.56 | |  | | 25 | 19 | 0.19 | |  | | <0.0001 | |
| Dizziness | 1 | 1 | 0.04 |  | | 2 | 2 | 0.08 |  | 0 | 0 | 0.00 |  | | 19 | 13 | 0.52 | |  | | 22 | 16 | 0.16 | |  | | <0.0001 | |
| Headache | 0 | 0 | 0.00 |  | | 0 | 0 | 0.00 |  | 1 | 1 | 0.04 |  | | 0 | 0 | 0.00 | |  | | 1 | 1 | 0.01 | |  | | 0.7457 | |
| Hypoaesthesia | 0 | 0 | 0.00 |  | | 1 | 1 | 0.04 |  | 0 | 0 | 0.00 |  | | 1 | 1 | 0.04 | |  | | 2 | 2 | 0.02 | |  | | 0.6207 | |
| Musculoskeletal and connective tissue disorders | 6 | 6 | 0.23 |  | | 1 | 1 | 0.04 |  | 0 | 0 | 0.00 |  | | 0 | 0 | 0.00 | |  | | 7 | 7 | 0.07 | |  | | 0.0054 | |
| Back pain | 1 | 1 | 0.04 |  | | 0 | 0 | 0.00 |  | 0 | 0 | 0.00 |  | | 0 | 0 | 0.00 | |  | | 1 | 1 | 0.01 | |  | | 1.0000 | |
| Arthralgia | 2 | 2 | 0.08 |  | | 0 | 0 | 0.00 |  | 0 | 0 | 0.00 |  | | 0 | 0 | 0.00 | |  | | 2 | 2 | 0.02 | |  | | 0.2499 | |
| Myalgia | 1 | 1 | 0.04 |  | | 0 | 0 | 0.00 |  | 0 | 0 | 0.00 |  | | 0 | 0 | 0.00 | |  | | 1 | 1 | 0.01 | |  | | 1.0000 | |
| Coccydynia | 1 | 1 | 0.04 |  | | 0 | 0 | 0.00 |  | 0 | 0 | 0.00 |  | | 0 | 0 | 0.00 | |  | | 1 | 1 | 0.01 | |  | | 1.0000 | |
| Pain in extremity | 1 | 1 | 0.04 |  | | 1 | 1 | 0.04 |  | 0 | 0 | 0.00 |  | | 0 | 0 | 0.00 | |  | | 2 | 2 | 0.02 | |  | | 1.0000 | |
| Infections and infestations | 230 | 215 | 8.41 |  | | 143 | 143 | 5.72 |  | 12 | 12 | 0.48 |  | | 180 | 176 | 7.04 | |  | | 565 | 546 | 5.43 | |  | | <0.0001 | |
| Nasopharyngitis | 0 | 0 | 0.00 |  | | 1 | 1 | 0.04 |  | 1 | 1 | 0.04 |  | | 6 | 6 | 0.24 | |  | | 8 | 8 | 0.08 | |  | | 0.0091 | |
| Rhinitis | 0 | 0 | 0.00 |  | | 1 | 1 | 0.04 |  | 0 | 0 | 0.00 |  | | 1 | 1 | 0.04 | |  | | 2 | 2 | 0.02 | |  | | 0.6207 | |
| Pharyngitis | 2 | 2 | 0.08 |  | | 6 | 6 | 0.24 |  | 0 | 0 | 0.00 |  | | 0 | 0 | 0.00 | |  | | 8 | 8 | 0.08 | |  | | 0.0052 | |
| Tonsillitis | 2 | 2 | 0.08 |  | | 7 | 7 | 0.28 |  | 0 | 0 | 0.00 |  | | 0 | 0 | 0.00 | |  | | 9 | 9 | 0.09 | |  | | 0.0016 | |
| Gastroenteritis viral | 1 | 1 | 0.04 |  | | 0 | 0 | 0.00 |  | 0 | 0 | 0.00 |  | | 0 | 0 | 0.00 | |  | | 1 | 1 | 0.01 | |  | | 1.0000 | |
| Gastroenteritis | 2 | 1 | 0.04 |  | | 8 | 8 | 0.32 |  | 1 | 1 | 0.04 |  | | 0 | 0 | 0.00 | |  | | 11 | 10 | 0.10 | |  | | 0.0014 | |
| Conjunctivitis | 0 | 0 | 0.00 |  | | 1 | 1 | 0.04 |  | 0 | 0 | 0.00 |  | | 0 | 0 | 0.00 | |  | | 1 | 1 | 0.01 | |  | | 0.7457 | |
| Periodontitis | 0 | 0 | 0.00 |  | | 0 | 0 | 0.00 |  | 0 | 0 | 0.00 |  | | 1 | 1 | 0.04 | |  | | 1 | 1 | 0.01 | |  | | 0.7457 | |
| Influenza | 1 | 1 | 0.04 |  | | 0 | 0 | 0.00 |  | 0 | 0 | 0.00 |  | | 0 | 0 | 0.00 | |  | | 1 | 1 | 0.01 | |  | | 1.0000 | |
| Upper respiratory tract infection | 222 | 210 | 8.21 |  | | 119 | 119 | 4.76 |  | 9 | 9 | 0.36 |  | | 172 | 168 | 6.72 | |  | | 522 | 506 | 5.03 | |  | | <0.0001 | |
| Bronchitis | 0 | 0 | 0.00 |  | | 0 | 0 | 0.00 |  | 1 | 1 | 0.04 |  | | 0 | 0 | 0.00 | |  | | 1 | 1 | 0.01 | |  | | 0.7457 | |
| Metabolism and nutrition disorders | 1 | 1 | 0.04 |  | | 0 | 0 | 0.00 |  | 0 | 0 | 0.00 |  | | 0 | 0 | 0.00 | |  | | 1 | 1 | 0.01 | |  | | 1.0000 | |
| Calcium deficiency | 1 | 1 | 0.04 |  | | 0 | 0 | 0.00 |  | 0 | 0 | 0.00 |  | | 0 | 0 | 0.00 | |  | | 1 | 1 | 0.01 | |  | | 1.0000 | |
| Cardiac disorders | 0 | 0 | 0.00 |  | | 0 | 0 | 0.00 |  | 1 | 1 | 0.04 |  | | 0 | 0 | 0.00 | |  | | 1 | 1 | 0.01 | |  | | 0.7457 | |
| Angina pectoris | 0 | 0 | 0.00 |  | | 0 | 0 | 0.00 |  | 1 | 1 | 0.04 |  | | 0 | 0 | 0.00 | |  | | 1 | 1 | 0.01 | |  | | 0.7457 | |
| Vascular disorders | 0 | 0 | 0.00 |  | | 0 | 0 | 0.00 |  | 0 | 0 | 0.00 |  | | 3 | 2 | 0.08 | |  | | 3 | 2 | 0.02 | |  | | 0.1853 | |
| Flushing | 0 | 0 | 0.00 |  | | 0 | 0 | 0.00 |  | 0 | 0 | 0.00 |  | | 3 | 2 | 0.08 | |  | | 3 | 2 | 0.02 | |  | | 0.1853 | |
| Blood and lymphatic system disorders | 2 | 2 | 0.08 |  | | 0 | 0 | 0.00 |  | 0 | 0 | 0.00 |  | | 0 | 0 | 0.00 | |  | | 2 | 2 | 0.02 | |  | | 0.2499 | |
| Lymphadenitis | 2 | 2 | 0.08 |  | | 0 | 0 | 0.00 |  | 0 | 0 | 0.00 |  | | 0 | 0 | 0.00 | |  | | 2 | 2 | 0.02 | |  | | 0.2499 | |
| Social circumstances | 0 | 0 | 0.00 |  | | 0 | 0 | 0.00 |  | 0 | 0 | 0.00 |  | | 1 | 1 | 0.04 | |  | | 1 | 1 | 0.01 | |  | | 0.7457 | |
| Hearing disability | 0 | 0 | 0.00 |  | | 0 | 0 | 0.00 |  | 0 | 0 | 0.00 |  | | 1 | 1 | 0.04 | |  | | 1 | 1 | 0.01 | |  | | 0.7457 | |
| Eye disorders | 0 | 0 | 0.00 |  | | 0 | 0 | 0.00 |  | 0 | 0 | 0.00 |  | | 1 | 1 | 0.04 | |  | | 1 | 1 | 0.01 | |  | | 0.7457 | |
| Vision blurred | 0 | 0 | 0.00 |  | | 0 | 0 | 0.00 |  | 0 | 0 | 0.00 |  | | 1 | 1 | 0.04 | |  | | 1 | 1 | 0.01 | |  | | 0.7457 | |
| Immune system disorders | 1 | 1 | 0.04 |  | | 0 | 0 | 0.00 |  | 0 | 0 | 0.00 |  | | 0 | 0 | 0.00 | |  | | 1 | 1 | 0.01 | |  | | 1.0000 | |
| Hypersensitivity | 1 | 1 | 0.04 |  | | 0 | 0 | 0.00 |  | 0 | 0 | 0.00 |  | | 0 | 0 | 0.00 | |  | | 1 | 1 | 0.01 | |  | | 1.0000 | |
| Injury, poisoning and surgical complications | 1 | 1 | 0.04 |  | | 1 | 1 | 0.04 |  | 0 | 0 | 0.00 |  | | 0 | 0 | 0.00 | |  | | 2 | 2 | 0.02 | |  | | 1.0000 | |
| Injury | 1 | 1 | 0.04 |  | | 1 | 1 | 0.04 |  | 0 | 0 | 0.00 |  | | 0 | 0 | 0.00 | |  | | 2 | 2 | 0.02 | |  | | 1.0000 | |
| Injury, poisoning and procedural complications | 0 | 0 | 0.00 |  | | 1 | 1 | 0.04 |  | 0 | 0 | 0.00 |  | | 0 | 0 | 0.00 | |  | | 1 | 1 | 0.01 | |  | | 0.7457 | |
| Contusion | 0 | 0 | 0.00 |  | | 1 | 1 | 0.04 |  | 0 | 0 | 0.00 |  | | 0 | 0 | 0.00 | |  | | 1 | 1 | 0.01 | |  | | 0.7457 | |

Note：Unsolicited adverse events are coded using MedDRA (Version 23.1).

Table 8.2.1.1.3 Incidence and frequency of vaccination-related adverse events (by solicited and unsolicited)

|  | **Baoji City**  **(N=2557)** | | |  | **Hanzhong City**  **(N=2500)** | | |  | **Xianyang City**  **(N=2500)** | | |  | **Yan’an City**  **(N=2500)** | | |  | **Total**  **(N=10057)** | | |  | **Fisher P** |
| --- | --- | --- | --- | --- | --- | --- | --- | --- | --- | --- | --- | --- | --- | --- | --- | --- | --- | --- | --- | --- | --- |
| **Adverse event term** | **No. of events** | **No. of subjects** | **Incidence rate(%)** |  | **No. of events** | **No. of subjects** | **Incidence rate(%)** |  | **No. of events** | **No. of subjects** | **Incidence rate(%)** |  | **No. of events** | **No. of subjects** | **Incidence rate(%)** |  | **No. of events** | **No. of subjects** | **Incidence rate(%)** |  |  |
| **Solicited** | 73 | 67 | 2.62 |  | 21 | 19 | 0.76 |  | 47 | 37 | 1.48 |  | 86 | 51 | 2.04 |  | 227 | 174 | 1.73 |  | <0.0001 |
| **Local adverse event** | 57 | 57 | 2.23 |  | 8 | 8 | 0.32 |  | 43 | 35 | 1.40 |  | 52 | 37 | 1.48 |  | 160 | 137 | 1.36 |  | <0.0001 |
| General disorders and administration site conditions | 57 | 57 | 2.23 |  | 8 | 8 | 0.32 |  | 43 | 35 | 1.40 |  | 52 | 37 | 1.48 |  | 160 | 137 | 1.36 |  | <0.0001 |
| Vaccination site rash | 3 | 3 | 0.12 |  | 0 | 0 | 0.00 |  | 0 | 0 | 0.00 |  | 10 | 7 | 0.28 |  | 13 | 10 | 0.10 |  | 0.0026 |
| Vaccination site pain | 30 | 30 | 1.17 |  | 5 | 5 | 0.20 |  | 19 | 19 | 0.76 |  | 17 | 17 | 0.68 |  | 71 | 71 | 0.71 |  | 0.0003 |
| Vaccination site swelling | 3 | 3 | 0.12 |  | 0 | 0 | 0.00 |  | 4 | 4 | 0.16 |  | 11 | 10 | 0.40 |  | 18 | 17 | 0.17 |  | 0.0033 |
| Vaccination site pruritus | 7 | 7 | 0.27 |  | 0 | 0 | 0.00 |  | 7 | 6 | 0.24 |  | 4 | 4 | 0.16 |  | 18 | 17 | 0.17 |  | 0.0379 |
| Injection site erythema | 14 | 14 | 0.55 |  | 2 | 2 | 0.08 |  | 10 | 10 | 0.40 |  | 3 | 3 | 0.12 |  | 29 | 29 | 0.29 |  | 0.0034 |
| Injection site induration | 0 | 0 | 0.00 |  | 1 | 1 | 0.04 |  | 3 | 3 | 0.12 |  | 7 | 6 | 0.24 |  | 11 | 10 | 0.10 |  | 0.0216 |
| **Systemic adverse event** | 16 | 11 | 0.43 |  | 13 | 11 | 0.44 |  | 4 | 4 | 0.16 |  | 34 | 25 | 1.00 |  | 67 | 51 | 0.51 |  | 0.0005 |
| Gastrointestinal Disorders | 6 | 5 | 0.20 |  | 4 | 3 | 0.12 |  | 0 | 0 | 0.00 |  | 21 | 14 | 0.56 |  | 31 | 22 | 0.22 |  | 0.0001 |
| Nausea | 4 | 4 | 0.16 |  | 2 | 2 | 0.08 |  | 0 | 0 | 0.00 |  | 16 | 13 | 0.52 |  | 22 | 19 | 0.19 |  | 0.0001 |
| Vomiting | 2 | 2 | 0.08 |  | 1 | 1 | 0.04 |  | 0 | 0 | 0.00 |  | 3 | 3 | 0.12 |  | 6 | 6 | 0.06 |  | 0.4402 |
| Diarrhoea | 0 | 0 | 0.00 |  | 1 | 1 | 0.04 |  | 0 | 0 | 0.00 |  | 2 | 1 | 0.04 |  | 3 | 2 | 0.02 |  | 0.6207 |
| General disorders and administration site conditions | 4 | 4 | 0.16 |  | 6 | 6 | 0.24 |  | 2 | 2 | 0.08 |  | 6 | 6 | 0.24 |  | 18 | 18 | 0.18 |  | 0.4563 |
| Pyrexia | 4 | 4 | 0.16 |  | 6 | 6 | 0.24 |  | 2 | 2 | 0.08 |  | 4 | 4 | 0.16 |  | 16 | 16 | 0.16 |  | 0.5906 |
| Asthenia | 0 | 0 | 0.00 |  | 0 | 0 | 0.00 |  | 0 | 0 | 0.00 |  | 2 | 2 | 0.08 |  | 2 | 2 | 0.02 |  | 0.1853 |
| Skin and subcutaneous tissue disorders | 1 | 1 | 0.04 |  | 0 | 0 | 0.00 |  | 0 | 0 | 0.00 |  | 1 | 1 | 0.04 |  | 2 | 2 | 0.02 |  | 1.0000 |
| Mucocutaneous rash | 1 | 1 | 0.04 |  | 0 | 0 | 0.00 |  | 0 | 0 | 0.00 |  | 1 | 1 | 0.04 |  | 2 | 2 | 0.02 |  | 1.0000 |
| Respiratory, thoracic and mediastinal disorders | 3 | 3 | 0.12 |  | 0 | 0 | 0.00 |  | 0 | 0 | 0.00 |  | 2 | 2 | 0.08 |  | 5 | 5 | 0.05 |  | 0.1796 |
| Cough | 3 | 3 | 0.12 |  | 0 | 0 | 0.00 |  | 0 | 0 | 0.00 |  | 2 | 2 | 0.08 |  | 5 | 5 | 0.05 |  | 0.1796 |
| Nervous system disorders | 2 | 2 | 0.08 |  | 2 | 2 | 0.08 |  | 2 | 2 | 0.08 |  | 2 | 2 | 0.08 |  | 8 | 8 | 0.08 |  | 1.0000 |
| Headache | 2 | 2 | 0.08 |  | 2 | 2 | 0.08 |  | 2 | 2 | 0.08 |  | 2 | 2 | 0.08 |  | 8 | 8 | 0.08 |  | 1.0000 |
| Metabolism and nutrition disorders | 0 | 0 | 0.00 |  | 1 | 1 | 0.04 |  | 0 | 0 | 0.00 |  | 0 | 0 | 0.00 |  | 1 | 1 | 0.01 |  | 0.7457 |
| Decreased appetite | 0 | 0 | 0.00 |  | 1 | 1 | 0.04 |  | 0 | 0 | 0.00 |  | 0 | 0 | 0.00 |  | 1 | 1 | 0.01 |  | 0.7457 |
| Musculoskeletal and connective tissue disorders | 0 | 0 | 0.00 |  | 0 | 0 | 0.00 |  | 0 | 0 | 0.00 |  | 2 | 2 | 0.08 |  | 2 | 2 | 0.02 |  | 0.1853 |
| Myalgia | 0 | 0 | 0.00 |  | 0 | 0 | 0.00 |  | 0 | 0 | 0.00 |  | 2 | 2 | 0.08 |  | 2 | 2 | 0.02 |  | 0.1853 |
| **Unsolicited** | 29 | 28 | 1.10 |  | 4 | 4 | 0.16 |  | 2 | 2 | 0.08 |  | 19 | 15 | 0.60 |  | 54 | 49 | 0.49 |  | <0.0001 |
| **Systemic adverse event** | 29 | 28 | 1.10 |  | 4 | 4 | 0.16 |  | 2 | 2 | 0.08 |  | 19 | 15 | 0.60 |  | 54 | 49 | 0.49 |  | <0.0001 |
| Gastrointestinal Disorders | 2 | 2 | 0.08 |  | 0 | 0 | 0.00 |  | 0 | 0 | 0.00 |  | 2 | 2 | 0.08 |  | 4 | 4 | 0.04 |  | 0.3436 |
| Enteritis | 0 | 0 | 0.00 |  | 0 | 0 | 0.00 |  | 0 | 0 | 0.00 |  | 1 | 1 | 0.04 |  | 1 | 1 | 0.01 |  | 0.7457 |
| Abdominal pain | 1 | 1 | 0.04 |  | 0 | 0 | 0.00 |  | 0 | 0 | 0.00 |  | 1 | 1 | 0.04 |  | 2 | 2 | 0.02 |  | 1.0000 |
| Abdominal pain upper | 1 | 1 | 0.04 |  | 0 | 0 | 0.00 |  | 0 | 0 | 0.00 |  | 0 | 0 | 0.00 |  | 1 | 1 | 0.01 |  | 1.0000 |
| General disorders and administration site conditions | 0 | 0 | 0.00 |  | 0 | 0 | 0.00 |  | 0 | 0 | 0.00 |  | 2 | 2 | 0.08 |  | 2 | 2 | 0.02 |  | 0.1853 |
| Pyrexia | 0 | 0 | 0.00 |  | 0 | 0 | 0.00 |  | 0 | 0 | 0.00 |  | 1 | 1 | 0.04 |  | 1 | 1 | 0.01 |  | 0.7457 |
| Asthenia | 0 | 0 | 0.00 |  | 0 | 0 | 0.00 |  | 0 | 0 | 0.00 |  | 1 | 1 | 0.04 |  | 1 | 1 | 0.01 |  | 0.7457 |
| Skin and subcutaneous tissue disorders | 0 | 0 | 0.00 |  | 1 | 1 | 0.04 |  | 2 | 2 | 0.08 |  | 0 | 0 | 0.00 |  | 3 | 3 | 0.03 |  | 0.3389 |
| Urticaria | 0 | 0 | 0.00 |  | 0 | 0 | 0.00 |  | 1 | 1 | 0.04 |  | 0 | 0 | 0.00 |  | 1 | 1 | 0.01 |  | 0.7457 |
| Rash | 0 | 0 | 0.00 |  | 1 | 1 | 0.04 |  | 0 | 0 | 0.00 |  | 0 | 0 | 0.00 |  | 1 | 1 | 0.01 |  | 0.7457 |
| Dermatitis allergic | 0 | 0 | 0.00 |  | 0 | 0 | 0.00 |  | 1 | 1 | 0.04 |  | 0 | 0 | 0.00 |  | 1 | 1 | 0.01 |  | 0.7457 |
| Respiratory, thoracic and mediastinal disorders | 0 | 0 | 0.00 |  | 0 | 0 | 0.00 |  | 0 | 0 | 0.00 |  | 1 | 1 | 0.04 |  | 1 | 1 | 0.01 |  | 0.7457 |
| Dyspnoea | 0 | 0 | 0.00 |  | 0 | 0 | 0.00 |  | 0 | 0 | 0.00 |  | 1 | 1 | 0.04 |  | 1 | 1 | 0.01 |  | 0.7457 |
| Nervous system disorders | 1 | 1 | 0.04 |  | 2 | 2 | 0.08 |  | 0 | 0 | 0.00 |  | 11 | 11 | 0.44 |  | 14 | 14 | 0.14 |  | 0.0001 |
| Dizziness | 1 | 1 | 0.04 |  | 2 | 2 | 0.08 |  | 0 | 0 | 0.00 |  | 10 | 10 | 0.40 |  | 13 | 13 | 0.13 |  | 0.0004 |
| Hypoaesthesia | 0 | 0 | 0.00 |  | 0 | 0 | 0.00 |  | 0 | 0 | 0.00 |  | 1 | 1 | 0.04 |  | 1 | 1 | 0.01 |  | 0.7457 |
| Musculoskeletal and connective tissue disorders | 4 | 4 | 0.16 |  | 0 | 0 | 0.00 |  | 0 | 0 | 0.00 |  | 0 | 0 | 0.00 |  | 4 | 4 | 0.04 |  | 0.0156 |
| Back pain | 1 | 1 | 0.04 |  | 0 | 0 | 0.00 |  | 0 | 0 | 0.00 |  | 0 | 0 | 0.00 |  | 1 | 1 | 0.01 |  | 1.0000 |
| Arthralgia | 2 | 2 | 0.08 |  | 0 | 0 | 0.00 |  | 0 | 0 | 0.00 |  | 0 | 0 | 0.00 |  | 2 | 2 | 0.02 |  | 0.2499 |
| Myalgia | 1 | 1 | 0.04 |  | 0 | 0 | 0.00 |  | 0 | 0 | 0.00 |  | 0 | 0 | 0.00 |  | 1 | 1 | 0.01 |  | 1.0000 |
| Infections and infestations | 19 | 19 | 0.74 |  | 1 | 1 | 0.04 |  | 0 | 0 | 0.00 |  | 2 | 2 | 0.08 |  | 22 | 22 | 0.22 |  | <0.0001 |
| Pharyngitis | 0 | 0 | 0.00 |  | 1 | 1 | 0.04 |  | 0 | 0 | 0.00 |  | 0 | 0 | 0.00 |  | 1 | 1 | 0.01 |  | 0.7457 |
| Tonsillitis | 1 | 1 | 0.04 |  | 0 | 0 | 0.00 |  | 0 | 0 | 0.00 |  | 0 | 0 | 0.00 |  | 1 | 1 | 0.01 |  | 1.0000 |
| Upper respiratory tract infection | 18 | 18 | 0.70 |  | 0 | 0 | 0.00 |  | 0 | 0 | 0.00 |  | 2 | 2 | 0.08 |  | 20 | 20 | 0.20 |  | <0.0001 |
| Vascular disorders | 0 | 0 | 0.00 |  | 0 | 0 | 0.00 |  | 0 | 0 | 0.00 |  | 1 | 1 | 0.04 |  | 1 | 1 | 0.01 |  | 0.7457 |
| Flushing | 0 | 0 | 0.00 |  | 0 | 0 | 0.00 |  | 0 | 0 | 0.00 |  | 1 | 1 | 0.04 |  | 1 | 1 | 0.01 |  | 0.7457 |
| Blood and lymphatic system disorders | 2 | 2 | 0.08 |  | 0 | 0 | 0.00 |  | 0 | 0 | 0.00 |  | 0 | 0 | 0.00 |  | 2 | 2 | 0.02 |  | 0.2499 |
| Lymphadenitis | 2 | 2 | 0.08 |  | 0 | 0 | 0.00 |  | 0 | 0 | 0.00 |  | 0 | 0 | 0.00 |  | 2 | 2 | 0.02 |  | 0.2499 |
| Immune system disorders | 1 | 1 | 0.04 |  | 0 | 0 | 0.00 |  | 0 | 0 | 0.00 |  | 0 | 0 | 0.00 |  | 1 | 1 | 0.01 |  | 1.0000 |
| Hypersensitivity | 1 | 1 | 0.04 |  | 0 | 0 | 0.00 |  | 0 | 0 | 0.00 |  | 0 | 0 | 0.00 |  | 1 | 1 | 0.01 |  | 1.0000 |

Note：Unsolicited adverse events are coded using MedDRA (Version 23.1).

Table 8.2.1.1.4 Severity of vaccination-related adverse events (n(%)) (by solicited and unsolicited)

| **Adverse event term** | **Baoji City**  **(N=2557)** | | | |  | **Hanzhong City**  **(N=2500)** | | | |  | **Xianyang City**  **(N=2500)** | | | |  | **Yan’an City**  **(N=2500)** | | | | |  | | **Total**  **(N=10057)** | | | | |  | | **KW_P** | |  |
| --- | --- | --- | --- | --- | --- | --- | --- | --- | --- | --- | --- | --- | --- | --- | --- | --- | --- | --- | --- | --- | --- | --- | --- | --- | --- | --- | --- | --- | --- | --- | --- | --- |
|  | **Grade 1** | **Grade 2** | **Grade 3** | **Total** |  | **Grade 1** | **Grade 2** | **Grade 3** | **Total** |  | **Grade 1** | **Grade 2** | **Grade 3** | **Total** |  | | **Grade 1** | **Grade 2** | **Grade 3** | **Total** | |  | | **Grade 1** | **Grade 2** | **Grade 3** | **Total** | |  | |  | |
| Overall | 70(2.74) | 24(0.94) | 0(0.00) | 94(3.68) |  | 19(0.76) | 1(0.04) | 1(0.04) | 21(0.84) |  | 27(1.08) | 11(0.44) | 0(0.00) | 38(1.52) |  | | 29(1.16) | 24(0.96) | 8(0.32) | 61(2.44) | |  | | 145(1.44) | 60(0.60) | 9(0.09) | 214(2.13) | |  | | 0.0001 | |
| **Solicited** | 57(2.23) | 10(0.39) | 0(0.00) | 67(2.62) |  | 18(0.72) | 1(0.04) | 0(0.00) | 19(0.76) |  | 27(1.08) | 10(0.40) | 0(0.00) | 37(1.48) |  | | 29(1.16) | 16(0.64) | 6(0.24) | 51(2.04) | |  | | 131(1.30) | 37(0.37) | 6(0.06) | 174(1.73) | |  | | 0.0004 | |
| **Local adverse event** | 50(1.96) | 7(0.27) | 0(0.00) | 57(2.23) |  | 8(0.32) | 0(0.00) | 0(0.00) | 8(0.32) |  | 27(1.08) | 8(0.32) | 0(0.00) | 35(1.40) |  | | 27(1.08) | 6(0.24) | 4(0.16) | 37(1.48) | |  | | 112(1.11) | 21(0.21) | 4(0.04) | 137(1.36) | |  | | 0.1086 | |
| General disorders and administration site conditions | 50(1.96) | 7(0.27) | 0(0.00) | 57(2.23) |  | 8(0.32) | 0(0.00) | 0(0.00) | 8(0.32) |  | 27(1.08) | 8(0.32) | 0(0.00) | 35(1.40) |  | | 27(1.08) | 6(0.24) | 4(0.16) | 37(1.48) | |  | | 112(1.11) | 21(0.21) | 4(0.04) | 137(1.36) | |  | | 0.1086 | |
| Vaccination site rash | 0(0.00) | 3(0.12) | 0(0.00) | 3(0.12) |  | 0(0.00) | 0(0.00) | 0(0.00) | 0(0.00) |  | 0(0.00) | 0(0.00) | 0(0.00) | 0(0.00) |  | | 5(0.20) | 1(0.04) | 1(0.04) | 7(0.28) | |  | | 5(0.05) | 4(0.04) | 1(0.01) | 10(0.10) | |  | | 0.1306 | |
| Vaccination site pain | 26(1.02) | 4(0.16) | 0(0.00) | 30(1.17) |  | 5(0.20) | 0(0.00) | 0(0.00) | 5(0.20) |  | 11(0.44) | 8(0.32) | 0(0.00) | 19(0.76) |  | | 14(0.56) | 2(0.08) | 1(0.04) | 17(0.68) | |  | | 56(0.56) | 14(0.14) | 1(0.01) | 71(0.71) | |  | | 0.0670 | |
| Vaccination site swelling | 3(0.12) | 0(0.00) | 0(0.00) | 3(0.12) |  | 0(0.00) | 0(0.00) | 0(0.00) | 0(0.00) |  | 4(0.16) | 0(0.00) | 0(0.00) | 4(0.16) |  | | 8(0.32) | 1(0.04) | 1(0.04) | 10(0.40) | |  | | 15(0.15) | 1(0.04) | 1(0.01) | 17(0.17) | |  | | 0.4753 | |
| Vaccination site pruritus | 7(0.27) | 0(0.00) | 0(0.00) | 7(0.27) |  | 0(0.00) | 0(0.00) | 0(0.00) | 0(0.00) |  | 6(0.24) | 0(0.00) | 0(0.00) | 6(0.24) |  | | 2(0.08) | 2(0.08) | 0(0.00) | 4(0.16) | |  | | 15(0.15) | 2(0.02) | 0(0.00) | 17(0.17) | |  | | 0.0312 | |
| Injection site erythema | 14(0.55) | 0(0.00) | 0(0.00) | 14(0.55) |  | 2(0.08) | 0(0.00) | 0(0.00) | 2(0.08) |  | 10(0.40) | 0(0.00) | 0(0.00) | 10(0.40) |  | | 2(0.08) | 0(0.00) | 1(0.04) | 3(0.12) | |  | | 28(0.28) | 0(0.00) | 1(0.01) | 29(0.29) | |  | | 0.0341 | |
| Injection site induration | 0(0.00) | 0(0.00) | 0(0.00) | 0(0.00) |  | 1(0.04) | 0(0.00) | 0(0.00) | 1(0.04) |  | 3(0.12) | 0(0.00) | 0(0.00) | 3(0.12) |  | | 6(0.24) | 0(0.00) | 0(0.00) | 6(0.24) | |  | | 10(0.10) | 0(0.00) | 0(0.00) | 10(0.10) | |  | | 1.0000 | |
| **Systemic adverse event** | 7(0.27) | 4(0.16) | 0(0.00) | 11(0.43) |  | 10(0.40) | 1(0.04) | 0(0.00) | 11(0.44) |  | 2(0.08) | 2(0.08) | 0(0.00) | 4(0.16) |  | | 11(0.44) | 12(0.48) | 2(0.08) | 25(1.00) | |  | | 30(0.30) | 19(0.19) | 2(0.02) | 51(0.51) | |  | | 0.0624 | |
| Gastrointestinal Disorders | 4(0.16) | 1(0.04) | 0(0.00) | 5(0.20) |  | 3(0.12) | 0(0.00) | 0(0.00) | 3(0.12) |  | 0(0.00) | 0(0.00) | 0(0.00) | 0(0.00) |  | | 7(0.28) | 7(0.28) | 0(0.00) | 14(0.56) | |  | | 14(0.14) | 8(0.08) | 0(0.00) | 22(0.22) | |  | | 0.1957 | |
| Nausea | 4(0.16) | 0(0.00) | 0(0.00) | 4(0.16) |  | 2(0.08) | 0(0.00) | 0(0.00) | 2(0.08) |  | 0(0.00) | 0(0.00) | 0(0.00) | 0(0.00) |  | | 7(0.28) | 6(0.24) | 0(0.00) | 13(0.52) | |  | | 13(0.13) | 6(0.06) | 0(0.00) | 19(0.19) | |  | | 0.1470 | |
| Vomiting | 1(0.04) | 1(0.04) | 0(0.00) | 2(0.08) |  | 1(0.04) | 0(0.00) | 0(0.00) | 1(0.04) |  | 0(0.00) | 0(0.00) | 0(0.00) | 0(0.00) |  | | 2(0.08) | 1(0.04) | 0(0.00) | 3(0.12) | |  | | 4(0.04) | 2(0.02) | 0(0.00) | 6(0.06) | |  | | 0.7316 | |
| Diarrhoea | 0(0.00) | 0(0.00) | 0(0.00) | 0(0.00) |  | 1(0.04) | 0(0.00) | 0(0.00) | 1(0.04) |  | 0(0.00) | 0(0.00) | 0(0.00) | 0(0.00) |  | | 0(0.00) | 1(0.04) | 0(0.00) | 1(0.04) | |  | | 1(0.01) | 1(0.01) | 0(0.00) | 2(0.02) | |  | | NA | |
| General disorders and administration site conditions | 2(0.08) | 2(0.08) | 0(0.00) | 4(0.16) |  | 5(0.20) | 1(0.04) | 0(0.00) | 6(0.24) |  | 0(0.00) | 2(0.08) | 0(0.00) | 2(0.08) |  | | 2(0.08) | 3(0.12) | 1(0.04) | 6(0.24) | |  | | 9(0.09) | 8(0.08) | 1(0.01) | 18(0.18) | |  | | 0.1756 | |
| Pyrexia | 2(0.08) | 2(0.08) | 0(0.00) | 4(0.16) |  | 5(0.20) | 1(0.04) | 0(0.00) | 6(0.24) |  | 0(0.00) | 2(0.08) | 0(0.00) | 2(0.08) |  | | 2(0.08) | 2(0.08) | 0(0.00) | 4(0.16) | |  | | 9(0.09) | 7(0.07) | 0(0.00) | 16(0.16) | |  | | 0.2400 | |
| Asthenia | 0(0.00) | 0(0.00) | 0(0.00) | 0(0.00) |  | 0(0.00) | 0(0.00) | 0(0.00) | 0(0.00) |  | 0(0.00) | 0(0.00) | 0(0.00) | 0(0.00) |  | | 0(0.00) | 1(0.04) | 1(0.04) | 2(0.08) | |  | | 0(0.00) | 1(0.01) | 1(0.01) | 2(0.02) | |  | | NA | |
| Skin and subcutaneous tissue disorders | 0(0.00) | 1(0.04) | 0(0.00) | 1(0.04) |  | 0(0.00) | 0(0.00) | 0(0.00) | 0(0.00) |  | 0(0.00) | 0(0.00) | 0(0.00) | 0(0.00) |  | | 0(0.00) | 1(0.04) | 0(0.00) | 1(0.04) | |  | | 0(0.00) | 2(0.02) | 0(0.00) | 2(0.02) | |  | | NA | |
| Mucocutaneous rash | 0(0.00) | 1(0.04) | 0(0.00) | 1(0.04) |  | 0(0.00) | 0(0.00) | 0(0.00) | 0(0.00) |  | 0(0.00) | 0(0.00) | 0(0.00) | 0(0.00) |  | | 0(0.00) | 1(0.04) | 0(0.00) | 1(0.04) | |  | | 0(0.00) | 2(0.02) | 0(0.00) | 2(0.02) | |  | | NA | |
| Respiratory, thoracic and mediastinal disorders | 3(0.12) | 0(0.00) | 0(0.00) | 3(0.12) |  | 0(0.00) | 0(0.00) | 0(0.00) | 0(0.00) |  | 0(0.00) | 0(0.00) | 0(0.00) | 0(0.00) |  | | 1(0.04) | 1(0.04) | 0(0.00) | 2(0.08) | |  | | 4(0.04) | 1(0.01) | 0(0.00) | 5(0.05) | |  | | 0.2207 | |
| Cough | 3(0.12) | 0(0.00) | 0(0.00) | 3(0.12) |  | 0(0.00) | 0(0.00) | 0(0.00) | 0(0.00) |  | 0(0.00) | 0(0.00) | 0(0.00) | 0(0.00) |  | | 1(0.04) | 1(0.04) | 0(0.00) | 2(0.08) | |  | | 4(0.04) | 1(0.01) | 0(0.00) | 5(0.05) | |  | | 0.2207 | |
| Nervous system disorders | 2(0.08) | 0(0.00) | 0(0.00) | 2(0.08) |  | 2(0.08) | 0(0.00) | 0(0.00) | 2(0.08) |  | 2(0.08) | 0(0.00) | 0(0.00) | 2(0.08) |  | | 2(0.08) | 0(0.00) | 0(0.00) | 2(0.08) | |  | | 8(0.08) | 0(0.00) | 0(0.00) | 8(0.08) | |  | | 1.0000 | |
| Headache | 2(0.08) | 0(0.00) | 0(0.00) | 2(0.08) |  | 2(0.08) | 0(0.00) | 0(0.00) | 2(0.08) |  | 2(0.08) | 0(0.00) | 0(0.00) | 2(0.08) |  | | 2(0.08) | 0(0.00) | 0(0.00) | 2(0.08) | |  | | 8(0.08) | 0(0.00) | 0(0.00) | 8(0.08) | |  | | 1.0000 | |
| Metabolism and nutrition disorders | 0(0.00) | 0(0.00) | 0(0.00) | 0(0.00) |  | 1(0.04) | 0(0.00) | 0(0.00) | 1(0.04) |  | 0(0.00) | 0(0.00) | 0(0.00) | 0(0.00) |  | | 0(0.00) | 0(0.00) | 0(0.00) | 0(0.00) | |  | | 1(0.01) | 0(0.00) | 0(0.00) | 1(0.01) | |  | | NA | |
| Decreased appetite | 0(0.00) | 0(0.00) | 0(0.00) | 0(0.00) |  | 1(0.04) | 0(0.00) | 0(0.00) | 1(0.04) |  | 0(0.00) | 0(0.00) | 0(0.00) | 0(0.00) |  | | 0(0.00) | 0(0.00) | 0(0.00) | 0(0.00) | |  | | 1(0.01) | 0(0.00) | 0(0.00) | 1(0.01) | |  | | NA | |
| Musculoskeletal and connective tissue disorders | 0(0.00) | 0(0.00) | 0(0.00) | 0(0.00) |  | 0(0.00) | 0(0.00) | 0(0.00) | 0(0.00) |  | 0(0.00) | 0(0.00) | 0(0.00) | 0(0.00) |  | | 0(0.00) | 1(0.04) | 1(0.04) | 2(0.08) | |  | | 0(0.00) | 1(0.01) | 1(0.01) | 2(0.02) | |  | | NA | |
| Myalgia | 0(0.00) | 0(0.00) | 0(0.00) | 0(0.00) |  | 0(0.00) | 0(0.00) | 0(0.00) | 0(0.00) |  | 0(0.00) | 0(0.00) | 0(0.00) | 0(0.00) |  | | 0(0.00) | 1(0.04) | 1(0.04) | 2(0.08) | |  | | 0(0.00) | 1(0.01) | 1(0.01) | 2(0.02) | |  | | NA | |
| **Unsolicited** | 13(0.51) | 15(0.59) | 0(0.00) | 28(1.10) |  | 3(0.12) | 0(0.00) | 1(0.04) | 4(0.16) |  | 1(0.04) | 1(0.04) | 0(0.00) | 2(0.08) |  | | 0(0.00) | 13(0.52) | 2(0.08) | 15(0.60) | |  | | 17(0.17) | 29(0.29) | 3(0.03) | 49(0.49) | |  | | 0.0077 | |
| **Systemic adverse event** | 13(0.51) | 15(0.59) | 0(0.00) | 28(1.10) |  | 3(0.12) | 0(0.00) | 1(0.04) | 4(0.16) |  | 1(0.04) | 1(0.04) | 0(0.00) | 2(0.08) |  | | 0(0.00) | 13(0.52) | 2(0.08) | 15(0.60) | |  | | 17(0.17) | 29(0.29) | 3(0.03) | 49(0.49) | |  | | 0.0077 | |
| Gastrointestinal Disorders | 1(0.04) | 1(0.04) | 0(0.00) | 2(0.08) |  | 0(0.00) | 0(0.00) | 0(0.00) | 0(0.00) |  | 0(0.00) | 0(0.00) | 0(0.00) | 0(0.00) |  | | 0(0.00) | 2(0.08) | 0(0.00) | 2(0.08) | |  | | 1(0.01) | 3(0.03) | 0(0.00) | 4(0.04) | |  | | 0.3173 | |
| Enteritis | 0(0.00) | 0(0.00) | 0(0.00) | 0(0.00) |  | 0(0.00) | 0(0.00) | 0(0.00) | 0(0.00) |  | 0(0.00) | 0(0.00) | 0(0.00) | 0(0.00) |  | | 0(0.00) | 1(0.04) | 0(0.00) | 1(0.04) | |  | | 0(0.00) | 1(0.01) | 0(0.00) | 1(0.01) | |  | | NA | |
| Abdominal pain | 0(0.00) | 1(0.04) | 0(0.00) | 1(0.04) |  | 0(0.00) | 0(0.00) | 0(0.00) | 0(0.00) |  | 0(0.00) | 0(0.00) | 0(0.00) | 0(0.00) |  | | 0(0.00) | 1(0.04) | 0(0.00) | 1(0.04) | |  | | 0(0.00) | 2(0.02) | 0(0.00) | 2(0.02) | |  | | NA | |
| Abdominal pain upper | 1(0.04) | 0(0.00) | 0(0.00) | 1(0.04) |  | 0(0.00) | 0(0.00) | 0(0.00) | 0(0.00) |  | 0(0.00) | 0(0.00) | 0(0.00) | 0(0.00) |  | | 0(0.00) | 0(0.00) | 0(0.00) | 0(0.00) | |  | | 1(0.01) | 0(0.00) | 0(0.00) | 1(0.01) | |  | | NA | |
| General disorders and administration site conditions | 0(0.00) | 0(0.00) | 0(0.00) | 0(0.00) |  | 0(0.00) | 0(0.00) | 0(0.00) | 0(0.00) |  | 0(0.00) | 0(0.00) | 0(0.00) | 0(0.00) |  | | 0(0.00) | 1(0.04) | 1(0.04) | 2(0.08) | |  | | 0(0.00) | 1(0.01) | 1(0.01) | 2(0.02) | |  | | NA | |
| Pyrexia | 0(0.00) | 0(0.00) | 0(0.00) | 0(0.00) |  | 0(0.00) | 0(0.00) | 0(0.00) | 0(0.00) |  | 0(0.00) | 0(0.00) | 0(0.00) | 0(0.00) |  | | 0(0.00) | 1(0.04) | 0(0.00) | 1(0.04) | |  | | 0(0.00) | 1(0.01) | 0(0.00) | 1(0.01) | |  | | NA | |
| Asthenia | 0(0.00) | 0(0.00) | 0(0.00) | 0(0.00) |  | 0(0.00) | 0(0.00) | 0(0.00) | 0(0.00) |  | 0(0.00) | 0(0.00) | 0(0.00) | 0(0.00) |  | | 0(0.00) | 0(0.00) | 1(0.04) | 1(0.04) | |  | | 0(0.00) | 0(0.00) | 1(0.01) | 1(0.01) | |  | | NA | |
| Skin and subcutaneous tissue disorders | 0(0.00) | 0(0.00) | 0(0.00) | 0(0.00) |  | 0(0.00) | 0(0.00) | 1(0.04) | 1(0.04) |  | 1(0.04) | 1(0.04) | 0(0.00) | 2(0.08) |  | | 0(0.00) | 0(0.00) | 0(0.00) | 0(0.00) | |  | | 1(0.01) | 1(0.01) | 1(0.01) | 3(0.03) | |  | | 0.2207 | |
| Urticaria | 0(0.00) | 0(0.00) | 0(0.00) | 0(0.00) |  | 0(0.00) | 0(0.00) | 0(0.00) | 0(0.00) |  | 0(0.00) | 1(0.04) | 0(0.00) | 1(0.04) |  | | 0(0.00) | 0(0.00) | 0(0.00) | 0(0.00) | |  | | 0(0.00) | 1(0.01) | 0(0.00) | 1(0.01) | |  | | NA | |
| Rash | 0(0.00) | 0(0.00) | 0(0.00) | 0(0.00) |  | 0(0.00) | 0(0.00) | 1(0.04) | 1(0.04) |  | 0(0.00) | 0(0.00) | 0(0.00) | 0(0.00) |  | | 0(0.00) | 0(0.00) | 0(0.00) | 0(0.00) | |  | | 0(0.00) | 0(0.00) | 1(0.01) | 1(0.01) | |  | | NA | |
| Dermatitis allergic | 0(0.00) | 0(0.00) | 0(0.00) | 0(0.00) |  | 0(0.00) | 0(0.00) | 0(0.00) | 0(0.00) |  | 1(0.04) | 0(0.00) | 0(0.00) | 1(0.04) |  | | 0(0.00) | 0(0.00) | 0(0.00) | 0(0.00) | |  | | 1(0.01) | 0(0.00) | 0(0.00) | 1(0.01) | |  | | NA | |
| Respiratory, thoracic and mediastinal disorders | 0(0.00) | 0(0.00) | 0(0.00) | 0(0.00) |  | 0(0.00) | 0(0.00) | 0(0.00) | 0(0.00) |  | 0(0.00) | 0(0.00) | 0(0.00) | 0(0.00) |  | | 0(0.00) | 0(0.00) | 1(0.04) | 1(0.04) | |  | | 0(0.00) | 0(0.00) | 1(0.01) | 1(0.01) | |  | | NA | |
| Dyspnoea | 0(0.00) | 0(0.00) | 0(0.00) | 0(0.00) |  | 0(0.00) | 0(0.00) | 0(0.00) | 0(0.00) |  | 0(0.00) | 0(0.00) | 0(0.00) | 0(0.00) |  | | 0(0.00) | 0(0.00) | 1(0.04) | 1(0.04) | |  | | 0(0.00) | 0(0.00) | 1(0.01) | 1(0.01) | |  | | NA | |
| Nervous system disorders | 1(0.04) | 0(0.00) | 0(0.00) | 1(0.04) |  | 2(0.08) | 0(0.00) | 0(0.00) | 2(0.08) |  | 0(0.00) | 0(0.00) | 0(0.00) | 0(0.00) |  | | 0(0.00) | 9(0.36) | 2(0.08) | 11(0.44) | |  | | 3(0.03) | 9(0.09) | 2(0.02) | 14(0.14) | |  | | 0.0106 | |
| Dizziness | 1(0.04) | 0(0.00) | 0(0.00) | 1(0.04) |  | 2(0.08) | 0(0.00) | 0(0.00) | 2(0.08) |  | 0(0.00) | 0(0.00) | 0(0.00) | 0(0.00) |  | | 0(0.00) | 9(0.36) | 1(0.04) | 10(0.40) | |  | | 3(0.03) | 9(0.09) | 1(0.01) | 13(0.13) | |  | | 0.0076 | |
| Hypoaesthesia | 0(0.00) | 0(0.00) | 0(0.00) | 0(0.00) |  | 0(0.00) | 0(0.00) | 0(0.00) | 0(0.00) |  | 0(0.00) | 0(0.00) | 0(0.00) | 0(0.00) |  | | 0(0.00) | 0(0.00) | 1(0.04) | 1(0.04) | |  | | 0(0.00) | 0(0.00) | 1(0.01) | 1(0.01) | |  | | NA | |
| Musculoskeletal and connective tissue disorders | 2(0.08) | 2(0.08) | 0(0.00) | 4(0.16) |  | 0(0.00) | 0(0.00) | 0(0.00) | 0(0.00) |  | 0(0.00) | 0(0.00) | 0(0.00) | 0(0.00) |  | | 0(0.00) | 0(0.00) | 0(0.00) | 0(0.00) | |  | | 2(0.02) | 2(0.02) | 0(0.00) | 4(0.04) | |  | | NA | |
| Back pain | 0(0.00) | 1(0.04) | 0(0.00) | 1(0.04) |  | 0(0.00) | 0(0.00) | 0(0.00) | 0(0.00) |  | 0(0.00) | 0(0.00) | 0(0.00) | 0(0.00) |  | | 0(0.00) | 0(0.00) | 0(0.00) | 0(0.00) | |  | | 0(0.00) | 1(0.01) | 0(0.00) | 1(0.01) | |  | | NA | |
| Arthralgia | 2(0.08) | 0(0.00) | 0(0.00) | 2(0.08) |  | 0(0.00) | 0(0.00) | 0(0.00) | 0(0.00) |  | 0(0.00) | 0(0.00) | 0(0.00) | 0(0.00) |  | | 0(0.00) | 0(0.00) | 0(0.00) | 0(0.00) | |  | | 2(0.02) | 0(0.00) | 0(0.00) | 2(0.02) | |  | | NA | |
| Myalgia | 0(0.00) | 1(0.04) | 0(0.00) | 1(0.04) |  | 0(0.00) | 0(0.00) | 0(0.00) | 0(0.00) |  | 0(0.00) | 0(0.00) | 0(0.00) | 0(0.00) |  | | 0(0.00) | 0(0.00) | 0(0.00) | 0(0.00) | |  | | 0(0.00) | 1(0.01) | 0(0.00) | 1(0.01) | |  | | NA | |
| Infections and infestations | 9(0.35) | 10(0.39) | 0(0.00) | 19(0.74) |  | 1(0.04) | 0(0.00) | 0(0.00) | 1(0.04) |  | 0(0.00) | 0(0.00) | 0(0.00) | 0(0.00) |  | | 0(0.00) | 2(0.08) | 0(0.00) | 2(0.08) | |  | | 10(0.10) | 12(0.12) | 0(0.00) | 22(0.22) | |  | | 0.2512 | |
| Pharyngitis | 0(0.00) | 0(0.00) | 0(0.00) | 0(0.00) |  | 1(0.04) | 0(0.00) | 0(0.00) | 1(0.04) |  | 0(0.00) | 0(0.00) | 0(0.00) | 0(0.00) |  | | 0(0.00) | 0(0.00) | 0(0.00) | 0(0.00) | |  | | 1(0.01) | 0(0.00) | 0(0.00) | 1(0.01) | |  | | NA | |
| Tonsillitis | 0(0.00) | 1(0.04) | 0(0.00) | 1(0.04) |  | 0(0.00) | 0(0.00) | 0(0.00) | 0(0.00) |  | 0(0.00) | 0(0.00) | 0(0.00) | 0(0.00) |  | | 0(0.00) | 0(0.00) | 0(0.00) | 0(0.00) | |  | | 0(0.00) | 1(0.01) | 0(0.00) | 1(0.01) | |  | | NA | |
| Upper respiratory tract infection | 9(0.35) | 9(0.35) | 0(0.00) | 18(0.70) |  | 0(0.00) | 0(0.00) | 0(0.00) | 0(0.00) |  | 0(0.00) | 0(0.00) | 0(0.00) | 0(0.00) |  | | 0(0.00) | 2(0.08) | 0(0.00) | 2(0.08) | |  | | 9(0.09) | 11(0.11) | 0(0.00) | 20(0.20) | |  | | 0.1888 | |
| Vascular disorders | 0(0.00) | 0(0.00) | 0(0.00) | 0(0.00) |  | 0(0.00) | 0(0.00) | 0(0.00) | 0(0.00) |  | 0(0.00) | 0(0.00) | 0(0.00) | 0(0.00) |  | | 0(0.00) | 1(0.04) | 0(0.00) | 1(0.04) | |  | | 0(0.00) | 1(0.01) | 0(0.00) | 1(0.01) | |  | | NA | |
| Flushing | 0(0.00) | 0(0.00) | 0(0.00) | 0(0.00) |  | 0(0.00) | 0(0.00) | 0(0.00) | 0(0.00) |  | 0(0.00) | 0(0.00) | 0(0.00) | 0(0.00) |  | | 0(0.00) | 1(0.04) | 0(0.00) | 1(0.04) | |  | | 0(0.00) | 1(0.01) | 0(0.00) | 1(0.01) | |  | | NA | |
| Blood and lymphatic system disorders | 0(0.00) | 2(0.08) | 0(0.00) | 2(0.08) |  | 0(0.00) | 0(0.00) | 0(0.00) | 0(0.00) |  | 0(0.00) | 0(0.00) | 0(0.00) | 0(0.00) |  | | 0(0.00) | 0(0.00) | 0(0.00) | 0(0.00) | |  | | 0(0.00) | 2(0.02) | 0(0.00) | 2(0.02) | |  | | NA | |
| Lymphadenitis | 0(0.00) | 2(0.08) | 0(0.00) | 2(0.08) |  | 0(0.00) | 0(0.00) | 0(0.00) | 0(0.00) |  | 0(0.00) | 0(0.00) | 0(0.00) | 0(0.00) |  | | 0(0.00) | 0(0.00) | 0(0.00) | 0(0.00) | |  | | 0(0.00) | 2(0.02) | 0(0.00) | 2(0.02) | |  | | NA | |
| Immune system disorders | 0(0.00) | 1(0.04) | 0(0.00) | 1(0.04) |  | 0(0.00) | 0(0.00) | 0(0.00) | 0(0.00) |  | 0(0.00) | 0(0.00) | 0(0.00) | 0(0.00) |  | | 0(0.00) | 0(0.00) | 0(0.00) | 0(0.00) | |  | | 0(0.00) | 1(0.01) | 0(0.00) | 1(0.01) | |  | | NA | |
| Hypersensitivity | 0(0.00) | 1(0.04) | 0(0.00) | 1(0.04) |  | 0(0.00) | 0(0.00) | 0(0.00) | 0(0.00) |  | 0(0.00) | 0(0.00) | 0(0.00) | 0(0.00) |  | | 0(0.00) | 0(0.00) | 0(0.00) | 0(0.00) | |  | | 0(0.00) | 1(0.01) | 0(0.00) | 1(0.01) | |  | | NA | |

Note：(1) Unsolicited adverse events are coded using MedDRA (Version 23.1).

(2) If a subject experiences a same adverse event multiple times, the one with highest severity grade is selected.

#### Adverse events within 30 minutes post vaccination

Table 8.2.1.2.1 Summary of adverse events within 30 minutes post vaccination (SS)

|  | **Baoji City**  **(N=2557)** | | |  | **Hanzhong City**  **(N=2500)** | | |  | **Xianyang City**  **(N=2500)** | | |  | **Yan’an City**  **(N=2500)** | | |  | **Total**  **(N=10057)** | | |  | **Fisher P** |
| --- | --- | --- | --- | --- | --- | --- | --- | --- | --- | --- | --- | --- | --- | --- | --- | --- | --- | --- | --- | --- | --- |
|  | **No. of events** | **No. of subjects** | **Incidence rate(%)** |  | **No. of events** | **No. of subjects** | **Incidence rate(%)** |  | **No. of events** | **No. of subjects** | **Incidence rate(%)** |  | **No. of events** | **No. of subjects** | **Incidence rate(%)** |  | **No. of events** | **No. of subjects** | **Incidence rate(%)** |  |  |
| Overall adverse events | 37 | 34 | 1.33 |  | 6 | 3 | 0.12 |  | 21 | 18 | 0.72 |  | 30 | 19 | 0.76 |  | 94 | 74 | 0.74 |  | <0.0001 |
| Vaccination-related | 37 | 34 | 1.33 |  | 6 | 3 | 0.12 |  | 21 | 18 | 0.72 |  | 30 | 19 | 0.76 |  | 94 | 74 | 0.74 |  | <0.0001 |
| Vaccination-unrelated | 0 | 0 | 0.00 |  | 0 | 0 | 0.00 |  | 0 | 0 | 0.00 |  | 0 | 0 | 0.00 |  | 0 | 0 | 0.00 |  | NA |

Note：(1)Vaccination-related refers to the relationship between adverse events and vaccines as ’possibly related’, ’highly likely related’ and ’definitely related’.

(2)Vaccination-related refers to the relationship between adverse events and vaccines as ’possibly unrelated’ and ’definitely unrelated’.

#### Adverse events within 0-14 days post vaccination

Table 8.2.1.3.1 Summary of adverse events within 0-14 days post vaccination (SS)

|  | **Baoji City**  **(N=2557)** | | |  | **Hanzhong City**  **(N=2500)** | | |  | **Xianyang City**  **(N=2500)** | | |  | **Yan’an City**  **(N=2500)** | | |  | **Total**  **(N=10057)** | | |  | **Fisher P** |
| --- | --- | --- | --- | --- | --- | --- | --- | --- | --- | --- | --- | --- | --- | --- | --- | --- | --- | --- | --- | --- | --- |
|  | **No. of events** | **No. of subjects** | **Incidence rate(%)** |  | **No. of events** | **No. of subjects** | **Incidence rate(%)** |  | **No. of events** | **No. of subjects** | **Incidence rate(%)** |  | **No. of events** | **No. of subjects** | **Incidence rate(%)** |  | **No. of events** | **No. of subjects** | **Incidence rate(%)** |  |  |
| Overall adverse events | 290 | 260 | 10.17 |  | 172 | 145 | 5.80 |  | 58 | 46 | 1.84 |  | 368 | 248 | 9.92 |  | 888 | 699 | 6.95 |  | <0.0001 |
| Vaccination-related | 102 | 94 | 3.68 |  | 25 | 21 | 0.84 |  | 48 | 37 | 1.48 |  | 104 | 60 | 2.40 |  | 279 | 212 | 2.11 |  | <0.0001 |
| Vaccination-unrelated | 188 | 170 | 6.65 |  | 147 | 126 | 5.04 |  | 10 | 9 | 0.36 |  | 264 | 204 | 8.16 |  | 609 | 509 | 5.06 |  | <0.0001 |

Note：(1)Vaccination-related refers to the relationship between adverse events and vaccines as ’possibly related’, ’highly likely related’ and ’definitely related’.

(2)Vaccination-related refers to the relationship between adverse events and vaccines as ’possibly unrelated’ and ’definitely unrelated’.

#### Adverse events within 15-30 days post vaccination

Table 8.2.1.4.1 Summary of adverse events within 15-30 days post vaccination (SS)

|  | **Baoji City**  **(N=2557)** | | |  | **Hanzhong City**  **(N=2500)** | | |  | **Xianyang City**  **(N=2500)** | | |  | **Yan’an City**  **(N=2500)** | | |  | **Total**  **(N=10057)** | | |  | **Fisher P** |
| --- | --- | --- | --- | --- | --- | --- | --- | --- | --- | --- | --- | --- | --- | --- | --- | --- | --- | --- | --- | --- | --- |
|  | **No. of events** | **No. of subjects** | **Incidence rate(%)** |  | **No. of events** | **No. of subjects** | **Incidence rate(%)** |  | **No. of events** | **No. of subjects** | **Incidence rate(%)** |  | **No. of events** | **No. of subjects** | **Incidence rate(%)** |  | **No. of events** | **No. of subjects** | **Incidence rate(%)** |  |  |
| Overall adverse events | 60 | 56 | 2.19 |  | 50 | 50 | 2.00 |  | 11 | 11 | 0.44 |  | 54 | 53 | 2.12 |  | 175 | 170 | 1.69 |  | <0.0001 |
| Vaccination-related | 0 | 0 | 0.00 |  | 0 | 0 | 0.00 |  | 1 | 1 | 0.04 |  | 1 | 1 | 0.04 |  | 2 | 2 | 0.02 |  | 0.6207 |
| Vaccination-unrelated | 60 | 56 | 2.19 |  | 50 | 50 | 2.00 |  | 10 | 10 | 0.40 |  | 53 | 52 | 2.08 |  | 173 | 168 | 1.67 |  | <0.0001 |

Note：(1)Vaccination-related refers to the relationship between adverse events and vaccines as ’possibly related’, ’highly likely related’ and ’definitely related’.

(2)Vaccination-related refers to the relationship between adverse events and vaccines as ’possibly unrelated’ and ’definitely unrelated’.

### Analysis of adverse event following immunization(AEFI)

Table 8.2.2.1 Incidence and frequency of AEFI

|  |  | **Total (N=287608)** | | |
| --- | --- | --- | --- | --- |
| **AEFI** |  | **No. of events** | **No. of subjects** | **Incidence rate(%)** |
| **Overall** |  | 71 | 57 | 0.0198 |
| General reactions |  | 50 | 38 | 0.0132 |
| General disorders and administration site conditions |  | 30 | 25 | 0.0087 |
| Vaccination site rash |  | 1 | 1 | 0.0003 |
| Vaccination site pain |  | 12 | 12 | 0.0042 |
| Vaccination site pruritus |  | 2 | 2 | 0.0007 |
| Vaccination site swelling |  | 5 | 5 | 0.0017 |
| Injection site erythema |  | 1 | 1 | 0.0003 |
| Injection site induration |  | 2 | 2 | 0.0007 |
| Pyrexia |  | 5 | 5 | 0.0017 |
| Peripheral swelling |  | 1 | 1 | 0.0003 |
| Chest pain |  | 1 | 1 | 0.0003 |
| Skin and subcutaneous tissue disorders |  | 1 | 1 | 0.0003 |
| Rash |  | 1 | 1 | 0.0003 |
| Respiratory, thoracic and mediastinal disorders |  | 1 | 1 | 0.0003 |
| Cough |  | 1 | 1 | 0.0003 |
| Nervous system disorders |  | 1 | 1 | 0.0003 |
| Dizziness |  | 1 | 1 | 0.0003 |
| Musculoskeletal and connective tissue disorders |  | 14 | 11 | 0.0038 |
| Arthralgia |  | 2 | 2 | 0.0007 |
| Muscular weakness |  | 8 | 8 | 0.0028 |
| Pain in extremity |  | 3 | 3 | 0.0010 |
| Musculoskeletal pain |  | 1 | 1 | 0.0003 |
| Infections and infestations |  | 1 | 1 | 0.0003 |
| Stomach flu |  | 1 | 1 | 0.0003 |
| Vascular disorders |  | 2 | 2 | 0.0007 |
| Flushing |  | 2 | 2 | 0.0007 |
| Abnormal reactions |  | 3 | 3 | 0.0010 |
| Gastrointestinal disorders |  | 1 | 1 | 0.0003 |
| Abdominal pain |  | 1 | 1 | 0.0003 |
| Nervous system disorders |  | 1 | 1 | 0.0003 |
| Hypoaesthesia |  | 1 | 1 | 0.0003 |
| Skin and subcutaneous tissue disorders |  | 1 | 1 | 0.0003 |
| Dermatitis allergic |  | 1 | 1 | 0.0003 |
| Coincidental reactions |  | 11 | 10 | 0.0035 |
| General disorders and administration site conditions |  | 1 | 1 | 0.0003 |
| Chest discomfort |  | 1 | 1 | 0.0003 |
| Gastrointestinal disorders |  | 1 | 1 | 0.0003 |
| Enteritis |  | 1 | 1 | 0.0003 |
| Respiratory, thoracic and mediastinal disorders |  | 1 | 1 | 0.0003 |
| Dyspnoea |  | 1 | 1 | 0.0003 |
| Infections and infestations |  | 8 | 8 | 0.0028 |
| Tonsillitis |  | 1 | 1 | 0.0003 |
| Gastroenteritis |  | 2 | 2 | 0.0007 |
| Upper respiratory tract infection |  | 5 | 5 | 0.0017 |
| Psychogenic reactions |  | 5 | 5 | 0.0017 |
| Psychiatric disorders |  | 5 | 5 | 0.0017 |
| Conversion disorder |  | 5 | 5 | 0.0017 |
| Undetermined reactions |  | 2 | 1 | 0.0003 |
| General disorders and administration site conditions |  | 1 | 1 | 0.0003 |
| Chest discomfort |  | 1 | 1 | 0.0003 |
| Respiratory, thoracic and mediastinal disorders |  | 1 | 1 | 0.0003 |
| Dyspnoea |  | 1 | 1 | 0.0003 |
